# Machine Learning–Driven Bioprospecting of Cholinergic Medicinal Plants for Putative Leads Against Butyrylcholinesterase Towards Alzheimer’s Care

**DOI:** 10.1155/bri/4503245

**Published:** 2026-08-31

**Authors:** Gideon Ampoma Gyebi, Onomeyimi O. Onesi, Saheed Sabiu

**Affiliations:** ^1^ Department of Biotechnology and Food Science, Faculty of Applied Sciences, Durban University of Technology, P.O. Box 1334, Durban 4000, South Africa, dut.ac.za; ^2^ Department of Biochemistry, Faculty of Science and Technology, Bingham University, Karu, Nigeria, binghamuni.edu.ng

**Keywords:** Alzheimer’s diseases, butyrylcholinesterase, machine learning, medicinal plants, molecular docking, molecular dynamics simulation

## Abstract

Butyrylcholinesterase (BChE) plays a key role in preserving appropriate cholinergic neurotransmission that is essentially altered in the brains of advanced Alzheimer’s disease (AD), hence a therapeutic target. This study employed a machine learning (ML) bioactivity predictive model to explore the chemical space of potential BChE inhibitors. A cheminformatics pipeline was explored to create a ML model for BChE inhibition using structural insights complemented with a comprehensive variance importance plot (VIP) and correlation matrix analysis. Specifically, a compiled library of 2179 secondary metabolites (SMs) from 50 Nigerian medicinal plants with reported cholinergic activity was investigated using the ML model. After which, molecular modelling was used to further screen the active SMs (827). The final predicted models demonstrated significant robustness, with a correlation coefficient of 0.8981. Molecular docking investigation of the 827 SMs identified the top five candidates based on their scores: three triterpenoids (adipedatol, lupenone and β‐amyrin), one steroid (9 (11)‐dehydroergosterol benzoate) and one flavonoid (tiliroside). These leads exhibited favourable ADMET properties and promising safety profiles. Among these five, lupenone (−48.78 kcal/mol), β‐amyrin (−49.19 kcal/mol) and adipedatol (−48.87 kcal/mol), from *Peltophorum pterocarpum, Bryophyllum pinnatum* and Alchornea laxiflora, respectively, were the most promising leads with significant binding free energy compared to decamethonium (−17.64 kcal/mol) and more favourable van der Waals, electrostatics and nonpolar solvation energetics. Furthermore, the binding of these leads resulted in optimised interaction profiles that preserved the structural integrity of the BChE and aligned well with desirable drug‐like characteristics. These findings position the leads as promising candidates for therapeutic applications targeting BChE for AD management, subject to further in vitro and in vivo validation investigations.

## 1. Introduction

Alzheimer’s disease (AD) is one of the most pressing global health issues of our time [1]. Over 35 million individuals around the globe suffer from AD [2], and as the population ages, it is projected that the number of cases will go up to 66 million by 2030 and 115 million by 2050, emphasising the disease’s increasing global burden [3]. The pathophysiology of AD involves aggregation of β‐amyloid (Aβ) deposits, tau hyperphosphorylation and decreased acetylcholine (ACh) levels [4, 5]. However, the most common rationale for the process of AD development is the cholinergic hypothesis, which directly contributes to cognitive decline [6]. Normally, both acetylcholinesterase (AChE) and butyrylcholinesterase (BChE) are capable of hydrolysing ACh; BChE is thought to play a small part in controlling the amount of ACh in the brain. On the contrary, BChE has been linked to drug metabolism and detoxification [7] and is intimately linked to disorders, such as hepatic adiposity, obesity and cardiovascular disease as well as lipoprotein metabolism [8]. As AD progresses, the amount of AChE in the brain decreases to 55%–67% of normal values, while the level of BChE rises to 120% of normal levels, suggesting that BChE is essential for ACh hydrolysis in the latter stages of AD. This shift in enzymatic dominance highlights BChE not merely as a secondary player but as a crucial therapeutic target, especially in later stages of the disease [9]. The development of cholinergic impairments in the neocortex and limbic structures, such as the hippocampus and amygdala, which result in behavioural and cognitive dysfunction, is facilitated by the ratio of BChE to AChE moving from 0.6 to as high as 1.1 [10]. There are currently just three cholinesterase inhibitors (rivastigmine, donepezil and galantamine) that are licenced for the treatment of AD [11], with none as specific inhibitors of BChE. The use of these cholinergic inhibitors often leads to dose‐limiting side effects, including bradycardia, gastrointestinal disturbances and muscle cramps [12]. Recent studies have also implicated BChE in Aβ plaque maturation, neuroinflammation and tau phosphorylation, suggesting that its inhibition could confer disease‐modifying benefits beyond symptomatic relief [13]. Therefore, inhibition of BChE may be an effective strategy without causing noticeable side effects [14] and, hence, an important target for the creation of innovative medications for AD [7].

Herbal remedies have been used for medical purposes throughout the world since the beginning of time [15]. Because of the rich neuroprotective secondary metabolites (SMs) in medicinal plants, it is commonly accepted that they have therapeutic benefits for disorders of the neurological system, including pain, stroke, epilepsy, AD and Parkinson’s disease (PD) [16, 17]. Medicinal plants play a pivotal role in the management of neurological disorders due to their diverse bioactive compounds and therapeutic potential [18]. They have been reported to preserve the neuronal chemicals in the brain via the inhibition of different receptors’ actions in distinct ways [19]. Africa has a wide variety of plants, and it also has one of the oldest medical systems in the world in its traditional medicine (ATM). Potential sources of medicinal components in this region include more than 45,000 endemic plant species, which make up 25% of the global flora [20].

Specifically, in Nigeria, the ethnobotanical knowledge represents a largely untapped reservoir of pharmacological potential. Nigeria possesses a rich biodiversity of medicinal plants, with over 7000 species of higher plants [21] and a deep‐rooted tradition of herbal medicine with several plants used traditionally for memory enhancement, calming effects and treatment of age‐related cognitive disorders and few reports on their neuroprotective, anti‐inflammatory, antioxidant and cholinesterase inhibitory activities [22]. Despite this abundance, there is still insufficient scientific research on the SMs of Nigerian medicinal herbs, specifically when it pertains to neurodegenerative illnesses. The use of cutting‐edge computational technologies, such as machine learning (ML), to methodically bioprospect the SMs of this plant species based on anticipated bioactivity presents a potential.

The development of novel BChE inhibitors is limited by several factors, including the structural complexity of BChE, selectivity, the blood–brain barrier (BBB), therapeutic resistance and safety and toxicological concerns [23]. Through the application of computational approaches, the conventional drug discovery process has been reshaped to enhance the accuracy of lead identification, reduce both expense and time, and narrow down the range of compounds requiring evaluation in ex vivo or in vitro systems [24]. Although these methods are mostly employed in the early stages of development and identification of novel candidates [25], some of the challenges faced in the later stages of drug development can be greatly reduced with the use of these methods [26]. By employing these methods, time and material wastage can be minimised by downsizing the scope of compounds to be further tested through other models, as opposed to the physical trial‐and‐error methods [27].

ML models, trained on large datasets of known bioactive compounds and their molecular targets, can predict the biological activity of new or uncharacterised molecules based on chemical fingerprints, molecular descriptors or structure–activity relationships (SARs) [28]. While there are few anticholinergic ML models currently, this is the first report of a BChE model developed for the screening of Nigerian medicinal plants with reported neuroprotective activity. In this study, we employed a ML‐driven bioprospecting framework to systematically explore cholinergic medicinal plants for potential BChE inhibitors. In order to identify intriguing scaffolds, a curated library of plant‐derived metabolites was analysed using descriptor‐based ML models and then computationally validated. The findings highlight the potential of integrating artificial intelligence with ethnomedicine to guide the discovery of novel leads for Alzheimer’s care.

## 2. Materials and Methods

### 2.1. Bioactivity Dataset and Exploratory Data Analysis

The structures of reported inhibitors of BChE were retrieved in the SMILES notation and IC_50_ values in nanometres from the ChEMBL available up until 2023. The IC_50_ values of the compounds were converted to pIC_50_ (a negative logarithmic scale) and expressed as −log10 (IC_50_) to produce a more consistent distribution of the IC_50_ data [29]. A SMILES validation pipeline was put in place for filtering out invalid structures and salts before featurisation in order to guarantee data quality. For molecular representation and featurisation, the RDKit library was implemented to process chemical structures. 2048‐bit Morgan fingerprints with a bond radius of two were employed to represent compounds. The molecular descriptors of Lipinski’s rule of five were computed for the compounds using the RDKit software. Lipinski’s rule states that a compound’s molecular weight should not be higher than 500 Da. The water–octanol partition coefficient (Log *p*) should be less than 5. The hydrogen bond donor and acceptor should not be greater than five and ten, respectively [30]. To evaluate the relationship between Lipinski’s descriptors and bioactivity (pIC_50_) values visually and categorise the compounds to be active or inactive, the Matplotlib and Seaborn programs were employed as earlier reported by Hu and Bajorath [31] and highlighted as follows:

 Active: ≤ 1000 nM
 Intermediate > 1000 nM and < 10,000 nM
 Inactive: ≥ 10,000 nM

### 2.2. Dataset Division, and Machine Architecture and Metadata Routing

An 80:20 ratio was employed to divide the downlead datasets into training and testing subsets. Predictive models were constructed using the training set, and their performance was evaluated using the test set. Because of its capacity for resilience to high‐dimensional noise, ability to handle high‐dimensional and heterogeneous molecular descriptor data and its potential to identify nonlinear SAR patterns, a Random Forest (RF) regressor was chosen as the base learner. RF has been successfully applied to classify AChE inhibitors, often outperforming other ML models [32, 33]. For each molecular feature type, an RF‐based model was constructed to establish the relationship between inhibitory activity and molecular fingerprints. During the training phase, sample weights (wiwi) were employed to mitigate any dataset bias or uneven distribution of activity values. By using the Scikit‐learn (v1.4+) metadata routing framework, we were able to explicitly feed sample_weight through the learning_curve and cross_val_score functions using the params argument. Two distinct steps of the two‐stage hyperparameter optimisation model tuning process were successfully carried out. Stage 2 used Bayesian optimisation using the Optuna framework after an initial Stage 1 search. To reduce the mean root mean squared error (RMSE) during a fivefold cross‐validation (KFold, shuffle = True), an objective function was established. In order to examine a narrowed search space, the Tree‐structured Parzen Estimator (TPE) sampler was used for 60 trials: The search space included *n*_estimators (200–1000), max_depth (None, 5–30), min_samples_split (2–20), min_samples_leaf (1–10) and max_features (sqrt, log2, 0.1–0.3). The objective function minimised the RMSE across a fold cross‐validation (KFold, shuffle = True). An applicability domain (APD) was set up by considering the chemical similarity between the training set and the test/screening molecules in an effort to guarantee the accuracy of the predictions. The training set’s 2048‐bit Morgan fingerprints were employed to fit a Nearest Neighbours model using the Jaccard (Tanimoto) metric. Compounds with a Tanimoto similarity to their closest training neighbour below a particular threshold (e.g. *T* = 0.25 or \0.60) were identified as being outside the APD. Several metrics were employed to evaluate the model’s performance: RMSE, Pearson’s correlation coefficient (rr), mean absolute error (MAE) and coefficient of determination (*R* ^2^). An external library of curated SMs was screened using the optimised model. SMILES validation and featurisation, pIC_50_ prediction, conversion to IC_50_ (nM) using the relationship IC_50_ = 10 − pIC50 × 109, and filtering by both a potency threshold (pIC50 ≥ 6.0) and the APD similarity constraint comprised the screening procedure.

### 2.3. Library Preparation

A library of compounds derived from medicinal plants from Nigerian flora with reported neurotherapeutic potential was compiled using literature mining approach. The search strategy involved querying web‐based databases, including PubChem, Web of Science (WoS), Google Scholar and Scopus. The databases were systematically searched to identify and extract relevant compound data. Specific keywords were employed to improve the retrieval of relevant data, including ‘neuroprotection’, ‘neuroprotective’ LC‐MS’, ‘GC‐MS’, ‘HPLC‐MS’ and ‘Chemical composition’ and Nigeria medicinal plants. The retrieved data were organised and compiled in a Microsoft Excel spreadsheet. This approach ensured a thorough and inclusive compilation of available information on SMs from Nigerian medicinal plants. The canonical SMILES format of the identified compounds was obtained from PubChem and ChEMBL.

### 2.4. Molecular Docking Studies

#### 2.4.1. Ligand Preparation

The SMs that were predicted to be active (pIC_50_ values of 6) by the model were subsequently screened against the human BChE using molecular docking analysis to fully comprehend the compound’s binding mechanisms. The SDF of SMs, along with the reference inhibitor, decamethonium, was retrieved from the PubChem database (https://www.pubchem.ncbi.nlm.nih.gov). Ligand structures were uploaded through the Open Babel interface within the software of the PyRx‐Python Prescription 0.8 tool [34] and energy‐minimised using the conjugate gradient descent algorithm with the Universal Force Field (UFF) parameters [35].

#### 2.4.2. Protein Preparation

The 3D cocrystallised structure of BChE in complex with decamethonium (PDB ID: 6EP4, sequence length: 529 and resolution: 2.30 Å) [36] was accessed from the Protein Data Bank website (http://www.rcsb.org). The structure was further processed in MGL‐AutoDockTools [37] by first deleting the bound native ligand and water molecules. Subsequently, missing hydrogen atoms were introduced, and partial atomic charges were assigned together with Kollman charges using the same software.

#### 2.4.3. Molecular Docking Protocol Validation

Before docking SMs against the active site of BChE, the native ligand (decamethonium) was re‐docked into the catalytic region of the protein, which was defined by mapping the region surrounding the experimentally bound native ligand with specified dimensions (Å): centre *x*, *y*, *z*, −20.23, −40.64, −23.74, and size *x*, *y*, *z*, 23.07, 23.67, and 22.31 to validate the molecular docking technique. The native‐extracted ligands were further superimposed with the decamethonium’s best‐docked conformation using BIOVIA Discovery Studio Visualizer 20.1.0, San Diego, CA, USA, 2020. Additionally, the interaction with significant active site catalytic residues was noted.

#### 2.4.4. Molecular Docking

Molecular docking investigation was conducted using AutoDock Vina which is implemented in PyRx 0.8. The ligands were docked into the binding site regions of the enzyme that was earlier determined from the validation exercise. The docking grid box was determined by centring on the cocrystallised ligand, which designates the enzyme’s experimentally verified binding pocket. To enable a guarantee that the docking search space encompassed the essential interactions in charge of ligand recognition and activity, the grid dimensions were determined to completely encompass all residues bordering the active site and catalytic gorge.

The binding scores of the reference standard (decamethonium) and SMs against the BChE enzyme were recorded and subsequently ranked. Molecular interactions between the hit compounds (compounds with lowest binding scores) were observed using BIOVIA Discovery Studio Visualizer 20.1.0, San Diego, CA, USA, 2020.

#### 2.4.5. Molecular Dynamics Simulation of Apo and Ligand‐Bound Complexes

A 100‐ns MD simulation was further performed to investigate the thermostability stability of the BChE‐bound complexes of the top five SMs with the most negative docking scores. The studies were performed using GROMACS 2019.2 [38, 39]. The topology parameters for water molecules, ions and protein amino acids were assigned using the CHARMM36 m force field [40], while the CHARMM‐GUI CGenFF tool [41] was used for ligand parametrisation. The systems were solvated in a cubic box with TIP3P water and 1 nm padding, supplemented with 0.154 M NaCl. Energy minimisation employed the steepest descent method with a maximum force of 100 kJ·mol^−1^·nm^−1^ over 100,000 steps. An NVT ensemble was initiated to stabilise temperature, volume and atom count, followed by a Berendsen barostat to maintain atmospheric pressure and equilibrate the system at 310 K using the V‐rescale method [42]. A 100 ns production run was conducted under the NVT ensemble. To preserve hydrogen‐associated bond lengths, the LINear Constraint Solver (LINCS) algorithm was employed during the simulation [43]. The Particle Mesh Ewald (PME) method was employed to compute the long‐range electrostatic interactions with a cutoff distance of 1.2 nm [44]. The leap‐frog integrator was employed to solve the equations of motion, using a 1 fs timestep during equilibration and 2 fs during the production phase [45]. The system was set to record the snapshot of the trajectory every 0.1 ns, resulting in a total of 1000 frames for the 100‐ns simulation.

#### 2.4.6. Calculations of Binding Free Energy (BFE) and Post‐MD Parameters of Lead SM

The MD trajectories were further processed using VMD scripts to investigate post‐MD structural parameters, including hydrogen bond count, root mean square deviation (RMSD), solvent‐accessible surface area (SASA), radius of gyration (Rg) and root mean square fluctuation (RMSF) [46]. The data generated for these structural parameters were further analysed and plotted in Microsoft Excel 365 (Microsoft Corporation, 2023). Also, the BFE of the lead SMs was computed using Molecular Mechanics Generalized Born Surface Area (MM‐GBSA) algorithm employed in the gmx_MMPBSA package [47]. The salt concentration parameter was fixed at 0.154 M, and the Generalized Born (GB) solvation model was applied with the setting igb = 5 [47, 48]. Additionally, the BFE decomposition was performed to assess the individual contributions of each amino acid to ligand binding.

## 3. Results

### 3.1. Model Creation

**FIGURE 1:**
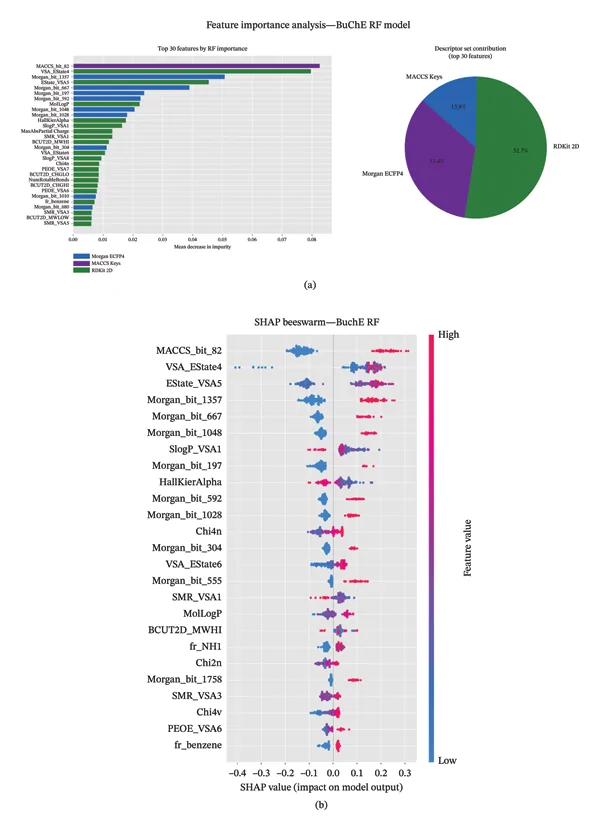
Model feature analysis. (a) Top 30 features, (b) descriptor set contribution and (c) SHAP beeswarms showing the SHAP contributions. The ‘Red’ dots (presence) for nearly each of these components are on the right side of the zero line, demonstrating that these chemical groups are crucial pharmacophores that boost inhibitory potency.

A total of 3852 reported BChE inhibitors were retrieved from the ChEMBL database, including their molecular notation, IC50 values (nM) and SMILES annotation. After removing entries outside the IC_50_ range, 40 compounds were excluded. About 3812 compounds having pIC50 values between 1.22 and 9.96 (mean ± SD: 5.70 ± 1.39) make up the final curated set. The descriptor set includes Morgan ECFP4 fingerprints (radius = 2, 2048 bits) widely regarded as the QSAR gold standard, MACCS structural keys (167 bits) and RDKit 2D physicochemical descriptors (214 continuous variables). After splitting, leakage audit checks for structurally identical or near‐identical compounds revealed that the test set included 765 compounds with pIC_50_ = 5.35 ± 1.28, whereas the training set had 3047 compounds with a mean potency of pIC_50_ = 5.79 ± 1.40. From the scaffold analysis, only 37 unique frameworks were identified in the test set compared to 1618 in the training set, suggesting that the evaluation set reflects a smaller scaffold space. Additionally, a Kolmogorov–Smirnov test demonstrated substantial changes in the pIC50 distributions between training and test sets (KS statistic = 0.1587, *p* < 0.0001), demonstrating that scaffold splitting separated compounds by both structure and activity. With 1392 inactive molecules (pIC50 < 5, 36.5%), 1035 moderate molecules (5–6, 27.2%) and 1385 active molecules (pIC_50_ ≥ 6, 36.3%), the BChE dataset showed a balanced distribution across activity classes. The significance of stratification for determining predictive accuracy was highlighted by a Kolmogorov–Smirnov test that indicated substantial differences between the train and test pIC_50_ distributions (*p* < 0.0001). The little observed imbalance was correct using inverse‐density sample weighting to the training set. To guarantee that minority regions participated proportionately during model training, the weighting strategy gave higher values to compounds with lower pIC_50_. The sample weights were standardised to ∼1.0 and varied from 0.37 to 76.18, indicating proper scaling (Figure 1). The results from fivefold nested cross‐validation revealed a low (mean RMSE = 0.2812) for the training error, while a larger cross‐validation error (mean CV RMSE = 0.7395 ± 0.0393) resulted in a 0.4583 generalisation gap, establishing a robust benchmark for subsequent model optimisation (Table 1).

**TABLE 1:** Fivefold nested cross‐validation results of the Random Forest model.

Fold	Train RMSE	CV RMSE	CV *R* ^2^	CV MAE
1	0.2844	0.7428	0.7378	0.5183
2	0.2817	0.7182	0.7097	0.5193
3	0.2754	0.8047	0.6808	0.5625
4	0.2766	0.7465	0.7234	0.5206
5	0.2877	0.6853	0.7470	0.5025
Mean	0.2812 ± 0.005	0.7395 ± 0.0393	0.7198 ± 0.0232	0.5246 ± 0.0201

**FIGURE 2:**
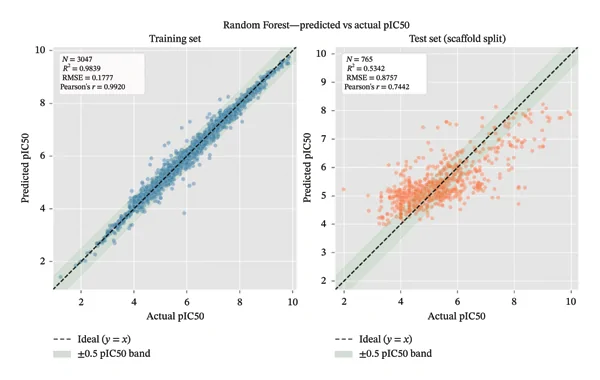
Scatter plot of experimental vs. predicted pIC50 values, of training set and test set of the BChE model.

The two‐stage optimisation involving Randomized Search CV (100 iterations × fivefold CV) and Optuna Bayesian Optimisation (60 trials × fivefold CV) of the RF model revealed strong performance on the BuChE dataset, with excellent fit to the training set (RMSE = 0.1777, MAE = 0.1029, *R* ^2^ = 0.9839, Pearson’s *r* = 0.992), where 98.4% of predictions fell within ±0.5 log units of experimental values. While scaffold‐based testing produced RMSE = 0.8757, MAE = 0.6828, *R* ^2^ = 0.5342 and Pearson’s *r* = 0.7442, with 76.9% of predictions within ±1.0 log units, cross‐validation validated generalisation with a mean CV RMSE of 0.7104 (Table 2 and Figure 2).

**TABLE 2:** The Random Forest model performance summary.

Set	*N*	RMSE	MAE	*R* ^2^	Pearson’s *r*	Within ±0.5	Within ±1.0
Training set	3047	0.1777	0.1209	0.9839	0.992	98.4%	99.7%
Fivefold CV (train)	3047	0.7104	—	—	—	—	—
Test set (scaffold)	765	0.8757	0.6828	0.5342	0.7442	45.5%	76.9%

**FIGURE 3:**
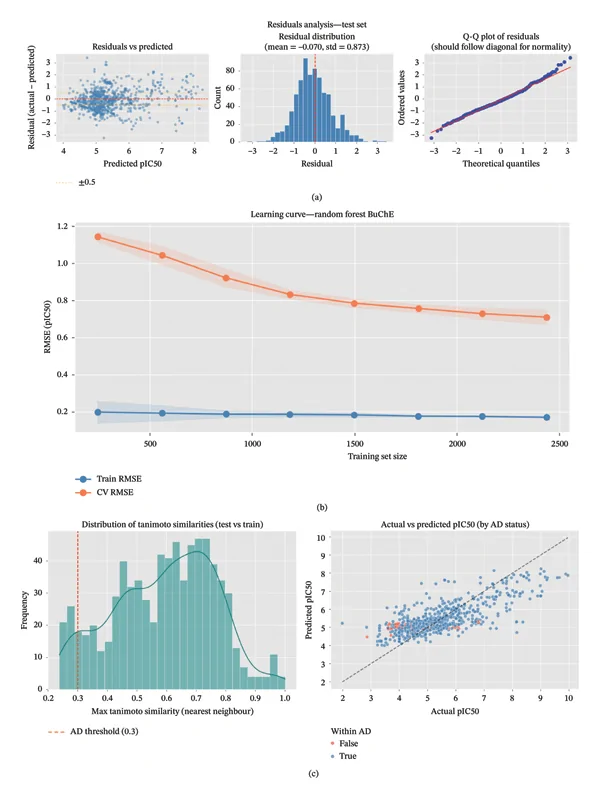
Exploratory data analysis of the model and inhibitor dataset: (a) residual analysis, (b) learning curve and (c) visualisation of applicability domain.

Prediction errors were generally well‐behaved, according to residual analysis of the test set (Figure 3). Many of the deviations in the residuals versus predicted a plot fell under ±0.5 log units, indicating no significant systematic bias. A balanced over‐ and underprediction throughout the activity range was demonstrated by the residual distribution, which was centred near zero (mean = −0.070, SD = 0.873). Predictive accuracy varied among activity classes, as determined by stratified performance analysis. The model returned RMSE = 0.8729 and MAE = 0.7140 for inactive compounds (*N* = 349) and RMSE = 0.5195 and MAE = 0.3940 for moderate compounds (*N* = 320). The prediction error for active compounds (*N* = 184) was higher (RMSE = 1.1842, MAE = 0.9877). While active compounds displayed partial correlation (*R* ^2^ = −0.5654), negative *R* ^2^ results for inactive and intermediate classes imply inadequate variance explanation within these categories. The learning curve indicated that model performance increases with larger datasets by showing that both training and cross‐validation RMSE reduced with increasing sample size. Most test compounds (712 of 765) were inside the specified similarity criterion (Tanimoto > 0.3), where the model attained RMSE = 0.869 and *R* ^2^ = 0.546, according to AD analysis. Predictions for the 53 compounds beyond the domain, on the contrary, showed inadequate variance explanation, with RMSE = 0.961 and negative *R* ^2^ (−0.052), confirming that the model offers robust predictions with its AD.

### 3.2. Features of the Model

The RF model’s feature analysis revealed that a mix of descriptor sets influenced prediction performance. RDKit 2D descriptors constructed the largest portion (52.7%) of the top 30 features, followed by MACCS Keys (13.8%) and Morgan RFP4 fingerprints (33.4%). MACCS structural keys, especially indices 45–49, were the most significant individual features. A deeper understanding of the factors influencing model was made possible by SHAP analysis. According to the beeswarm plot (Figure 1a), descriptors, such as MACCS structural bits, EState_VSA5, MolLogP and Morgan fingerprint features, had the biggest impact, with both high and low values having distinct effects on activity outcomes. The aggregate SHAP contributions revealed that MACCS Keys accounted for 13.2%, Morgan ECFP4 fingerprints for 34.7% of the explanatory power and RDKit 2D descriptors for 5.0%. Table 3 shows the structural fingerprints. The fingerprint fragments generated from SHAP demonstrate structural features that are consistently linked to predicted BuChE activity. A few of these bits match chemical groups that are known to affect cholinesterase binding.

**TABLE 3:** Structural fingerprints (local environments).

Bit ID	Chemical group	Likely structural fragment
MACCS_bit_82	C‐N‐C linkage	(Amine or amide)
Morgan_1357	Benzyl/phenyl‐alkyl	A benzene ring attached to a CH_2_ linker.
Morgan_667	Methoxy/ether	R−O−CH_3_ linkage.
Morgan_1048	Carbonyl/amide	C=O group within a ring or chain.
Morgan_197	Isopropyl/branched alkyl	−CH(CH_3_)_2_ or terminal methyls.
Morgan_592	Tertiary amine	*R*_3_ *N* *R*_3_ *N*(e.g. in a piperidine or diethylamine).
Morgan_1028	Substituted aromatic	Para‐ or metasubstituted phenyl ring.
Morgan_304	Heterocyclic nitrogen	Pyridine, piperidine or indole fragment.
Morgan_555	Halogenated aromatic	Cl, F or Br attached to a phenyl ring.
Morgan_1758	Aliphatic linker	Ethylene (−CH_2_−CH_2_−) or longer chains.

### 3.3. Physiochemical Properties

**FIGURE 4:**
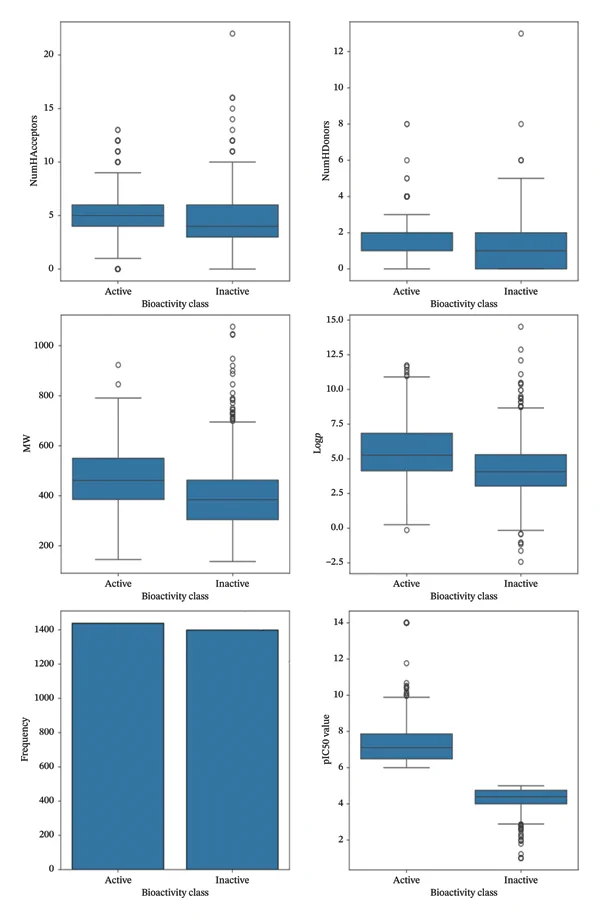
Exploratory data analysis of the curated BChE inhibitor dataset.

The graphical bar plots show that the number of predicted active compounds against BChE in the dataset was significantly more than that of the inactive compounds (Figure 2). Additionally, the molecular weights ranged from 380 to 550 Da for the active and 300 to 480 Da for the inactive compounds in the BChE model. The pIC_50_ values for the active molecules in the dataset were determined to range from 6.0 to 8.8, whereas the inactive molecules had a pIC_50_ value < 5. The log *p* values of the compounds ranged from approximately 4.0 to 6.5 for active molecules and 3.1 to 5.2 for inactive molecules. Chemical space analysis revealed that many of the active molecules in the curated training dataset followed Lipinski’s rule of five, suggesting they possess favourable drug‐like properties (Figure 4).

### 3.4. Screening of SMs From Nigerian Medicinal Plants

**FIGURE 5:**
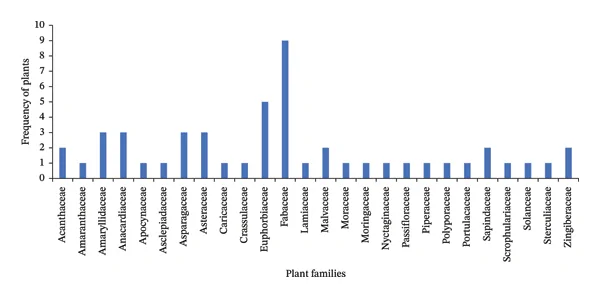
Distribution of Nigerian medicinal plants with cholinergic activity across different families.

Following a literature search of Nigerian plants with reported anticholinergic activity, 50 plants were identified. While the authors do not claim it was an exhaustive search, the purpose was to obtain SMs from a good number of plants with reported anticholinergic activity. The plant families and the specific plant parts showing anti‐BChE activity are listed in Supporting Table S1. The 50 Nigerian plants were distributed across 26 families. The families represented at least twice include Acanthaceae (2), Amaryllidaceae (3), Anacardiaceae (3), Asparagaceae (3), Asteraceae (3), Euphorbiaceae (5), Fabaceae (9), Malvaceae (2), Sapindaceae (2) and Zingiberaceae (2), with Fabaceae having the highest representation (Figure 5). A library of 2179 SMs from 50 Nigerian medicinal plants with reported neuroprotective activity was generated. Using the developed model, the compiled library of SMs was screened. From the 2179 compounds, the results revealed 827 compounds with predicted BChE activity. The pIC_50_ was between the active range values of 6.0 and 8.8, while a pIC_50_ value < 5 for the inactive molecules was observed.

### 3.5. Findings From Docking Analysis

**FIGURE 6:**
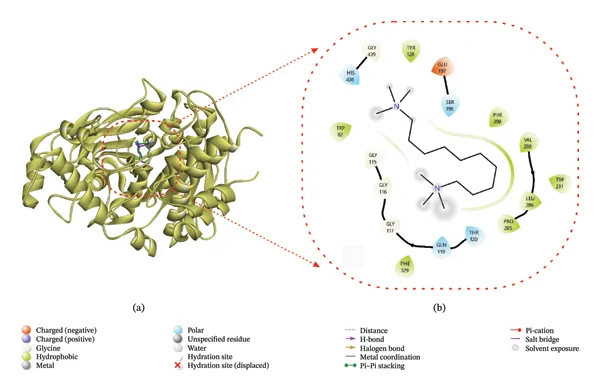
Validation of the molecular docking protocol: (a) a cartoon representation showing superimposition of the best‐docked conformer of the cocrystalised reference compound (decamethonium) on the extracted conformation (the red ligand colour represents the docked conformer, while the purple colour represents the extracted conformation); (b) the 2‐dimensional interactive plot of the docked conformation of decamethonium in the active site of BChE.

Validation of the molecular docking protocol is an important step to determine the accuracy, precision and reliability of the docking. The reference standard (decamethonium), as shown in Figure 4, was docked in a close alignment to the extracted native ligand and interacted with the residues of the binding site, such as Trp82, Glu197, Leu286 and Trp231. In order to visually check the similarities in their poses, the docked conformation of decamethonium with the lowest binding energy was superimposed over the cocrystalised ligand in the active pocket. The RMSD of 1.05 Å indicates that the native binding pose of decamethonium was reproduced with good fidelity (Figure 6). Following validation of the molecular docking protocol, the SMs (827) that demonstrated a predicted pIC_50_ value of ≥ 6 (IC_50_: 100–1000 nM) from the models were docked against the active site of BChE. The results of the docking scores from the docking analysis, including decamethonium and donepezil against hBChE, are presented in Table 4. The top 15 hits of SMs from the docking analyses to hBChE were selected based on their catalytic site interaction, orientation in the binding site and high negative binding scores. The binding scores of the 15 hit SMs ranged from −11.6 to −10.3 kcal/mol (Table 4). The results showed that donepezil and decamethonium were docked into the binding sites of the cocrystallised proteins with binding energies of −8.5 and −7.4 kcal/mol, respectively. From the top 15 hit SMs, the top six lead SMs were selected for amino acid interactive analysis and further molecular dynamics analysis. The leading SMs were tiliroside, 1,3,4‐trigalloyl‐beta‐D‐glucopyranose, β‐amyrin, lupenone, 9 (11)‐dehydroergosterol benzoate and adipedatol with binding scores of −11.6, −11.4, −11.4, −11.3, −11.2 and −11.2 kcal/mol, respectively. Notably, all top 15 SMs displayed higher negative binding scores when compared to decamethonium (−7.4 kcal/mol) and donepezil (−8.5 kcal/mol) (Table 2).

**TABLE 4:** Binding scores of top 15 secondary metabolites obtained from the docking analysis of compiled Nigeria medicinal plant against human butyrylcholinesterase.

	Compounds	Docking scores (kcal/mol)
S1	Decamethonium	−6.4
S2	Donepezil	−8.5
1	Tiliroside	**−11.6**
2	Lupenone	**−11.3**
3	β‐Amyrin	**−11.3**
4	9 (11)‐Dehydroergosterol benzoate	**−11.2**
5	Adipedatol	**−11.2**
6	1,3,4‐Trigalloyl‐beta‐D‐glucopyranose	**−11.0**
7	Olean‐12‐ene	−11.0
8	Octadecahydro‐2H‐picen‐3‐one	−11.1
9	3‐Acetyl‐11‐keto‐beta‐boswellic acid	−10.9
10	Rutin	−10.5
11	9,19‐Cyclolanostan‐3‐ol, acetate, (3.beta)	−10.4
12	Liriodenine	−10.4
13	Tannins	−10.4
14	Castalin	−10.3
15	Ellagic acid	−10.3

*Note:* S: standard; top 6 SMs are represented in bold fonts.

### 3.6. Interactions of the Top Six SMs With the Amino Acid in the Active Site of Human BChE

**FIGURE 7:**
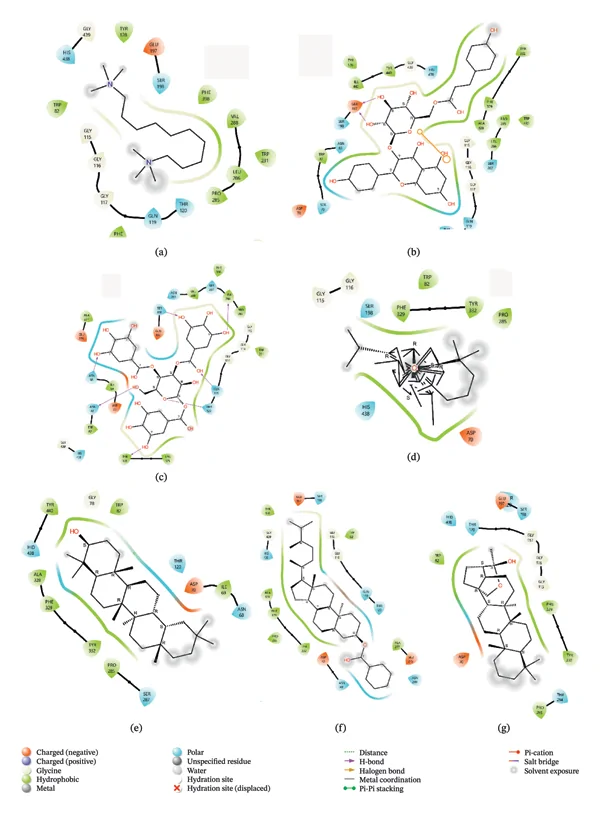
Interactive plots of the top six secondary metabolites and reference inhibitors from the docking analysis with amino acids in the active site of BChE: (i) 3D and (ii) 2D interaction. The ligands are displayed as sticks: (a) decamethonium, (b) tiliroside, (c) 1,3,4‐trigalloyl‐beta‐D‐glucopyranose, (d) lupenone, (e) beta‐amyrin, (f) 9 (11)‐dehydroergosterol benzoate and (g) adipedatol.

The amino acid interaction between the reference molecule and the top six lead SMs from the docking analysis is presented in Table 5. Most of the interactions between the ligand groups and the enzyme residues were hydrophobic. The native ligand (decamethonium) interacted via pi‐cation contact with Glu197, attractive charge contacts with Trp82, pi‐sigma with Trp82, pi‐alkyl with Trp231 and alkyl contact with Leu286. Carbon–hydrogen contacts were established with Tyr128, Gly115, Pro285 and Gln119 (Figure 7a). In a close docking pose as the reference compound, tiliroside was stabilised by 6 hydrogen bonds with residues Ser79, Ser198, Glu197, Gly119, Ser287 and His238; pi‐anion interaction with Asp70; pi‐pi stacking with Tyr332; and several van der Waals interactions (Figure 7b). 1,3,4‐Trigalloyl‐beta‐D‐glucopyranose formed 11 hydrogen bonds with residues Leu286, Gln119, Ans68, Asn289, Ser198, Thr120, Asn83, Tyr128, Gly115 and Glu197 and amide‐Pi stacking with Glu116 (Figure 7c). Lupenone was stabilised in the active site via pi‐sigma contact with Trp82, pi‐alkyl contacts with Trp82, Ala328, Phe329 and Tyr332; alkyl with Trp82; and several van der Waals (Figure 7d). Beta‐amyrin was stabilised in the active site through hydrogen bonds with Trp82 and mainly through hydrophobic interactions, including pi‐alkyl with Tyr332, Phe329 and Trp82, and alkyl contacts with Ala328 and His438 (Figure 7e). 9 (11)‐Dehydroergosterol benzoate did not form hydrogen bonds but interacted via pi‐alkyl contact with Phe329, Ala328, Tyr332 and Ala277 and alkyl contacts with His428 and Ala328 of hBChE (Figure 5f). Adipedatol formed a pi‐sigma bond with Tyr332, pi‐alkyl contacts with Phe329, Trp82 and Tyr332, and alkyl contacts with Pro285, Tyr332 and Trp52 (Figure 7g). Among the top six SMs, the top five SMs—9 (11)‐dehydroergosterol benzoate, lupenone, adipedatol, β‐amyrin and tiliroside—were selected for further evaluation based on their lower docking scores and interactions with the catalytic residues of BChE.

**TABLE 5:** Amino acid interaction of the top six docked secondary metabolites against butyrylcholinesterase.

Compounds	Hydrogen bonds	Hydrophobic interaction	Others
	Interacting residues	Interacting residues	
Donepezil	Tyr332	Pro285, Leu286, Trp231, Trp82, His438, Glu197, Met437, Trp430, Tyr440, Ala328	
Decamethonium		Trp82 Glu197 Leu286 Trp231	Tyr128 Gly115 Pro285 Gln119
9 (11)‐Dehydroergosterol benzoate		Phe329 Ala328 Trp82 His438 Tyr332 Ala277	
Tiliroside	His438 Glu197 Tyr128 Ser79 Asn83 Ser198 Gly117 Thr284 Ser287	Tyr332 Asp70	Trp82 Gly439 Gln119
1,3,4‐Trigalloyl‐beta‐D‐glucopyranose	Leu286, Ala119 Asn68 Glu275 Glu276 Thr284 Asn289 Ser287 GLn119 Thr120 Asn83 Gly115 Trp82 Tyr128 Glu197 Ser198 His438	Gl116	
Lupenone		Phe329 Ala328 Trp82 His438 Tyr332	Gly439 Gly116 Ser198 Thr120 Asp70
Adipedatol		Tyr332 Phe329 Trp82	Thr284 His438 Asp70 Gly115 Gly116 Gly117 Thr120 Ser198
Beta‐amyrin	Trp82	Phe329 Ala328 His438 Trp430 Tyr332	Gln119 Asn68 Ile69 Thr120 Gly116 Tyr440 Met437 Asp70 Pro285 Ser287

### 3.7. Pharmacokinetic and Absorption, Distribution, Metabolism, Excretion and Toxicity (ADMET) Properties of Top Five SMs

The five lead SMs identified by the docking analysis and subsequently examined using molecular dynamics were assessed in silico for predictive drug‐likeness and for ADMET analyses across an extensive array of filters and molecular descriptors. The results from these analyses are presented in Table 6. The molecular weight of the lead SM was between the range of 424.70 g/mol (lupenone) and 594.52 g/mol (tiliroside). Except for tiliroside with a large molecular weight and a high number of hydrogen bond donors/acceptors, all the lead SMs passed Lipinski’s rule of 5, suggesting that these compounds have favourable drug‐like properties. The human intestinal absorption (HIA) indicates the likelihood of a compound being absorbed in the human intestine, with values closer to 1 indicating better absorption. Lupenone (1.00) has the best chance of being absorbed in the human intestine, followed by β‐amyrin (0.91) and adipedatol (0.80). Tiliroside (0.127) and decamethonium (0.13) are likely to have poor absorption. All compounds are negative for being P‐glycoprotein substrates, which suggests they are not actively pumped out of cells by this protein. This is generally a good sign for absorption and distribution, as they are less likely to have their absorption hindered by P‐glycoprotein. The likelihood that the compound can cross the BBB was also evaluated. All compounds are likely to cross the BBB, with adipedatol (0.96) showing the highest probability, followed by lupenone (0.95) and β‐amyrin (0.94). Decamethonium has the lowest plasma protein binding (52.82%), suggesting that a larger fraction of it remains free and biologically active. However, the relatively high protein binding percentages of DHGB, tiliroside, lupenone, β‐amyrin and adipedatol may lower their effective concentration in plasma. Although DEM is a poor CYP450 1A2 inhibitor, it does not function as a substrate for the majority of enzymes, indicating that its total metabolic contribution may be minimal. Tiliroside is most likely to act as a substrate for CYP450 2C9, but it has moderate inhibition potential for CYP450 1A2 and 3A4, which may impact its metabolism. Lupenone and β‐amyrin adipedatol are nonsubstrates for several enzymes, indicating less metabolic interference. The half‐lives of all the SMs are relatively short (ranging from 1.74 h to 1.96 h). The difference in half‐lives is minimal, with lupenone having the longest half‐life (1.963 h), though it is only slightly longer than the others.

**TABLE 6:** In silico drug‐likeness and ADMET parameters of the top five secondary metabolites from the docking analysis to human BChE.

	DEM	DHGB	Tiliroside	Lupenone	β‐Amyrin	Adipedatol
Molecular weight (g/mol)	258.49	498.74	594.52	424.70	426.72	428.69
Num. heavy atoms	18	37	43	31	31	31
Num. arom. heavy atoms	0	6	22	0	0	0
Num. rotatable bonds	11	7	8	1	1	0
Num. H‐bond acceptors	0	2	13	1	1	2
Hydrogen bond donor	0	0	5	0	0	1
MLogP	3.52	9.12	3.76	6.82	6.92	6.36
Molar refractivity	83.77	156.64	149.51	134.18	134.88	29.46
TPSA (Å^2^)	0.00	26.30	216.58	17.07	20.23	216.58
Drug‐likeness						
Lipinski	Yes	Yes	No	Yes	Yes	Yes
Bioavailability score	0.55	0.55	0.55	0.55	0.55	0.55
Absorption (probability)						
HIA	HIA+ (0.13)	HIA+ (0.853)	HIA+ (0.127)	HIA+ (1.00)	HIA+ (0.91)	HIA+ (0.80)
P‐Glycoprotein substrate	Neg. (0.015)	Neg. (0.067)	Neg. (0.039)	Neg. (0.62)	Neg. (0.262)	Neg. (0.402)
Distribution (probability)						
Blood–brain barrier	BBB+ (0.76)	BBB+ (0.823)	BBB+ (0.84)	BBB+ (0.95)	BBB+ (0.94)	BBB+ (0.96)
PPB %	52.82	83.85	84.104	80.90	80.17	79.04
VD L/kg	0.52	−0.178	0.538	0.18	0.283	0.06
Metabolism (probability)						
CYP450 (1A2) substrate	Neg. (0.424)	Neg. (0.462)	Neg. (0.257)	Neg. (0.378)	Neg. (0.31)	Neg. (0.386)
CYP450 (1A2) inhibitor	Pos. (0.004)	Neg. (0.052)	Neg. (0.288)	Neg. (0.108)	Neg. (0.037)	Neg. (0.045)
CYP450 (3A4) substrate	Neg 0.066)	Neg. (0.689)	Neg. (0.372)	Neg. (0.644)	Pos. (0.576)	Pos. (0.544)
CYP450 (3A4) inhibitor	Neg. (0.002)	Neg. (0.306)	Neg. (0.615)	Neg. (0.202)	Neg. (0.089)	Neg. (0.097)
CYP450 (2C9) inhibitor	Neg. (0.003)	Neg. (0.365)	Neg. (0.398)	Neg. (0.126)	Neg. (0.034)	Neg. (0.09)
CYP450 (2C9) substrate	Neg. (0.414)	Pos. (0.311)	Pos. (0.469)	Pos. (0.447)	Pos. (0.347)	Pos. (0.341)
CYP450 (2C19) substrate	Neg. (0.17)	Neg. (0.363)	Neg. (0.406)	Neg. (0.546)	Neg. (0.426)	Neg. (0.497)
CYP450 (2C19) inhibitor	Neg. (0.011)	Neg. (0.261)	Neg. (0.268)	Neg. (0.172)	Neg. (0.149)	Neg. (0.336)
CYP450 (2D6) substrate	Neg. (0.62)	Neg. (0.354)	Neg. (0.345)	Neg. (0.241)	Neg. (0.404)	Neg. (0.312)
CYP450 (2D6) inhibitor	Neg. (0.076)	Neg. (0.448)	Neg. (0.432)	Neg. (0.263)	Neg. (0.321)	Neg. (0.339)
Elimination						
*T*_1/2_ (half‐life time)	1.783 h	1.903 h	1.744 h	1.963 h	1.878 h	1.828 h h
CL (clearance rate) mL/min/kg	2.151	0.978	1.24	1.07	1.25	1.408
Toxicity						
hERG blockers	Neg. (0.478)	Neg. (0.55)	Neg. (0.41)	Neg. (0.48)	Neg. (0.468)	Neg. (0.461)
H‐HT (human hepatotoxicity)	Neg. 0.042)	Neg. (0.5)	Neg. (0.224)	Neg. (0.086)	Neg. (0.06)	Neg. (0.152)
Carcinogen	Neg. (0.815)	Neg. (0.225)	Neg. (0.435)	Neg. (0.893)	Neg. (0.783)	Neg. (0.940)
LD_50_ (LD_50_ of acute toxicity)	2.478 ‐log mol/kg (859.905 mg/kg)	3.368 ‐log mol/kg (213.73 mg/kg)	2.56 ‐log mol/kg (871.672 mg/kg)	3.22 ‐log mol/kg (255.915 mg/kg)	3.285 ‐log mol/kg (221.387 mg/kg)	3.353 ‐log mol/kg (190.175 mg/kg
Synthetic accessibility	2.12	6.39	5.94	5.38	6.04	6.65

*Note:* DEM (decamethonium); DHGB (9 (11)‐dehydroergosterol benzoate).

Decamethonium has the highest clearance rate (2.151 mL/min/kg). DHGB has the lowest clearance rate (0.978 mL/min/kg), indicating slower removal and potentially sustained therapeutic effects. Adipedatol has a moderate clearance rate (1.408 mL/min/kg), positioning it between fast and slow elimination kinetics. With probability ranging from Neg. (0.41) for tiliroside (the least likely to block) to Neg. (0.55) for DHGB (slightly higher likelihood), all SMs are anticipated to be non‐hERG blockers. All the SMs were predicted not to be human hepatotoxic and carcinogenic. Decamethonium presented the highest synthetic accessibility score (2.12), suggesting ease of synthesis and potential cost‐effectiveness. The lowest synthetic accessibility (6.65) for adipedatol suggests that its synthesis is more complicated than for other SMs.

### 3.8. Thermodynamic BFE Analysis

Using the MM‐GBSA method, the BFE of the top five SMs and decamethonium to BChE was examined. The mean and standard deviation (SD) of the different energy components of the BFE of the top five SMs to BChE are presented in Table 7. The energetics of the BFE reveal that decamethonium was stabilised by a favourable balance of van der Waals interactions (−26.51 ± 5.26 kcal/mol) and electrostatic energy (−47.43 ± 32.76 kcal/mol) that compensated for the polar solvation energy term (40.53 ± 35.75 kcal/mol), suggesting that the ligand undergoes significant desolvation upon binding. With an additional stabilisation nonpolar solvation effect (−4.23 ± 0.88 kcal/mol), the overall BFE of decamethonium involves a combination of the gas phase and solvation contributions.

**TABLE 7:** The mean and SD of different energy components of the binding free energy of top docked secondary metabolites to BChE.

SYSTEMs	Δ_VDWAALS_	Δ_EEL_	ΔE_GB_	ΔE_SUR_	ΔG_GAS_	ΔG_SOLV_	Δ_TOTAL_
6EP4_DME	−26.51 ± 5.26	−47.43 ± 32.76	40.53 ± 35.75	−4.23 ± 0.88	−63.94 ± 36.58	56.3 ± 35.08	−17.64 ± 3.93
6EP4_DHGB	−38.81 ± 2.66	−12.46 ± 3.25	33.39 ± 3.02	−4.84 ± 0.28	−51.27 ± 3.88	28.55 ± 2.96	−22.72 ± 2.65
6EP4_tiliroside	−57.17 ± 5.93	−37.49 ± 11.48	67.18 ± 8.32	−7.59 ± 0.53	−94.66 ± 14.95	59.59 ± 8.00	−35.07 ± 8.40
6EP4_lupenone	−56.07 ± 2.73	−1.77 ± 2.24	14.95 ± 2.14	−5.88 ± 0.27	−57.85 ± 3.75	9.06 ± 2.09	−48.78 ± 3.14
6EP4_β‐amyrin	−54.51 ± 2.52	−6.82 ± 3.14	18.32 ± 1.79	−6.18 ± 0.20	−61.33 ± 3.56	12.13 ± 1.80	−49.19 ± 3.45
6EP4_adipedatol	−53.86 ± 2.14	−7.69 ± 2.64	18.56 ± 2.69	−5.87 ± 0.19	−61.56 ± 3.28	12.69 ± 2.66	−48.87 ± 2.94

*Note:* DME: decamethonium; DHGB; 9 (11)‐dehydroergosterol benzoate; Δ_VDWAALS_: change in van der Waals energy; Δ_EEL_: change in electrostatic energy; Δ_EGB_: change in electrostatic potential energy; Δ_ESUR_: change in surface energy; Δ_GGAS_: change in the Gibbs free energy of the gas phase; ΔG_SOLV_: change in Gibbs free energy of solvation; Δ_TOTAL_: total energy change.

The favourable BEF (−22.72 ± 2.65 kcal/mol) for the 6EP4_DHGB system reveals a contribution from van der Waals interactions (−38.81 ± 2.66 kcal/mol), electrostatic energy (−12.46 ± 3.25 kcal/mol) and nonpolar solvation energy (4.84 ± 0.28 kcal/mol) offsetting the unfavourable desolvation cost by polar solvation energy (33.39 ± 3.02 kcal/mol). This suggests that DHGB is likely to bind strongly to its target, mainly due to the favourable molecular interactions in the gas phase and with the nonpolar solvent.

The BFE of the 6EP4_tiliroside (−35.07 ± 8.40 kcal/mol) system was majorly stabilised by strong van der Waals interactions (−57.17 ± 5.93 kcal/mol) and minimal nonpolar solvation energy effect (−7.59 ± 0.53 kcal/mol) that compensated for the high polar solvation effects (67.18 ± 8.32 kcal/mol).

The individual component that makes up the favourable BFE (−35.07 ± 8.40 kcal/mol) of the 6EP4_lupenone system includes a balance of high van der Waals interactions (−56.07 ± 2.73 kcal/mol), very low electrostatic energy (−1.77 ± 2.24 kcal/mol) and nonpolar solvation energy (−5.88 ± 0.27 kcal/mol) terms that compensated for the moderate unfavourable polar solvation energy (9.06 ± 2.09 kcal/mol). Unlike the other SMs, the binding of lupenone in the binding pocket of BChE was majorly driving van der Waals interactions.

The total BFE (−49.19 ± 3.45 kcal/mol) for the 6EP4_β‐amyrin system was driven by the strong van der Waals interaction (−54.51 ± 2.52 kcal/mol), electrostatic energy (−6.82 ± 3.14 kcal/mol) and nonpolar solvation energy (−6.18 ± 0.20 kcal/mol) that counteracted the moderate destabilising polar solvation energy (12.13 ± 1.80 kcal/mol).

With one of the lowest overall BFE values (−49.19 kcal/mol) and good stabilisation in comparison with other systems, β‐amyrin is a promising option for additional study. Nonelectrostatic forces had the greatest impact on its binding, which is indicative of its hydrophobic characteristics and strong interaction dynamics.

The most significant contributor to the favourable binding energy of 6EP4_adipedatol (−48.87 ± 2.94 kcal/mol) is the van der Waals interactions with a large negative value of −53.86 kcal/mol, with moderate low electrostatic energy (−7.69 ± 2.64 kcal/mol) that compensated for the moderate destabilising nonpolar solvation energy (18.56 ± 2.69 kcal/mol). Overall, the highly negative total BFE (−48.87 ± 2.94 kcal/mol) highlights the strong and thermodynamically favourable nature of the interaction, making adipedatol a promising candidate for further study. This profile aligns closely with systems, such as β‐amyrin and lupenone, where strong van der Waals forces play a key role in stabilising the complex.

### 3.9. Postmolecular Structural Properties of Bound and Unbound BChE Systems

**FIGURE 8:**
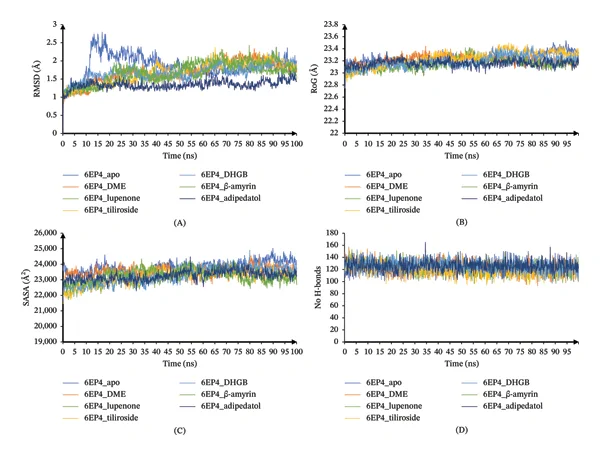
Analysis of MD trajectories of unbound protein, reference compound and top three secondary metabolites from the docking analysis to BChE: (a) the backbone‐root mean square deviation (RMSD) plots, (b) radius of gyration, (c) surface accessible surface area (SASA) and (d) variations in the number of H‐bonds (DME: decamethonium; DHGB; 9 (11)‐dehydroergosterol benzoate).

**FIGURE 9:**
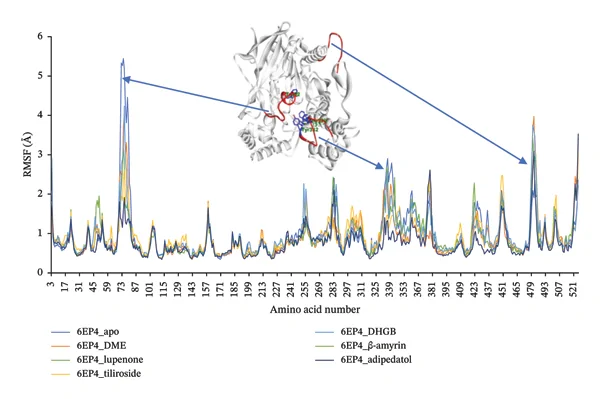
Per‐residue root mean square fluctuation (RMSF) plots of MD simulation of unbound protein, reference compound and top three secondary metabolites from the docking analysis to BChE. (DME: decamethonium; DHGB; 9 (11)‐dehydroergosterol benzoate).

The thermostability complexes of the cocrystalised ligand (decamethonium) and the top five SMs from the docking analysis were compared with the unbound protein during a full atomistic 100‐ns MD simulation. Table 8 presents the averages and SDs of all the parameters, while the plots of the thermodynamic parameters are presented in Figures 8 and 9. As observed from the RMSD plots, all the seven systems were equilibrated before 10 ns. While the complexes maintained a stable progression throughout the simulation with minimal fluctuations, the unbound protein demonstrated high fluctuations after equilibration (10 ns) until 40 ns. All the systems were all covered at the end of the simulation (Figure 8a). The mean RMSD value (1.85 ± 0.32 Å) for the unbound system serves as the baseline and suggests a relatively higher degree of structural flexibility in the absence of ligands. The high fluctuations that were experienced across this time frame may have caused the high mean RMSD value (1.85 ± 0.32 Å), as compared to the complexes. The RMSD values of all ligand‐bound complexes were lower than those of the unbound structure, suggesting that ligand interaction increases structural stability. The ligand‐bound systems presented RMSD values ranging from 1.62 Å to 1.69 Å, all lower than the apo form. This indicates that these ligands induce a more stable conformation in BChE upon binding. With the shortest SD (0.12 Å) and the lowest RMSD value (1.34 Å), adipedatol appears to interact more effectively with the protein, potentially inducing a more stable conformation and suggesting a very robust and reliable binding relationship with BChE. The RMSF plot shows maximum fluctuation around amino acid residues 327–343. The active site residues are outside this range (Figure 9). In a similar trend as the RMSD parameter, the unbound proteins presented a higher mean RMSF value (0.95 ± 0.67 Å), indicating a relatively higher level of flexibility in the absence of ligands. The adipedatol‐bound system (0.77 ± 0.39 Å) had the lowest RMSF value, implying the most significant reduction in flexibility and the strongest stabilising effect among all the ligands tested. Compared to the reference standard, 6EP4_DME (0.87 ± 0.53 Å) and the β‐amyrin‐bound system (0.87 ± 0.52 Å) presented the same RMSF values, signifying a notable reduction in protein flexibility. The 6EP4_TbG, 6EP4_DHGB and 6EP4_tiliroside systems show comparable RMSF values, suggesting that their impact on protein flexibility is relatively moderate and similar across this group. Adipedatol, with the lowest RMSF, further supports its strong stabilising effects observed in the RMSD analysis, making it a standout ligand in terms of promoting structural stability.

**TABLE 8:** The mean and standard deviation of thermodynamic parameters analysed from the MDS trajectories of top docked compounds complexed with BChE.

Mean	RMSD (Å)	RMSF (Å)	RoG (Å)	SASA (Å)	H‐bond
6EP4_Apo	1.85 ± 0.32	0.95 ± 0.67	23.23 ± 0.08	23799.9 ± 371.9	123.91 ± 8.65
6EP4_DME	1.68 ± 0.29	0.87 ± 0.53	23.21 ± 0.06	23542.7 ± 296.1	122.36 ± 9.47
6EP4_TbG	1.68 ± 0.29	0.92 ± 0.43	23.24 ± 0.12	23200.6 ± 454.3	119.56 ± 9.33
6EP4_DHGB	1.68 ± 0.20	0.92 ± 0.50	23.18 ± 0.09	23264.5 ± 513.8	125.34 ± 9.18
Tiliroside	1.68 ± 0.29	0.92 ± 0.43	23.24 ± 0.12	23200.6 ± 454.3	119.96 ± 9.33
Lupenone	1.62 ± 0.27	0.90 ± 0.50	23.16 ± 0.09	23163.8 ± 466.5	121.81 ± 9.52
β‐Amyrin	1.69 ± 0.28	0.87 ± 0.52	23.13 ± 0.07	23244.4 ± 354.7	122.39 ± 9.29
Adipedatol	1.34 ± 0.12	0.77 ± 0.39	23.16 ± 0.05	23263.2 ± 317.9	125.86 ± 8.41

*Note:* DME: decamethonium; DHGB; 9 (11)‐dehydroergosterol benzoate.

With the lowest RMSF, adipedatol stands out as a ligand that promotes structural stability because of its strong stabilising effects as seen in the RMSD analysis. Noteworthily, the important binding site residue (Tpr82) that is implicated in the catalysis of the BChE enzyme was around the region with the highest fluctuation. The analysis of the RMSF values of Tpr82 of the unbound proteins (1.272 Å), decamethonium_BChE (0.973), DHGB_BChE (0.822 Å), tiliroside_BChE (1.067 Å), lupenone_BChE (0.852 Å), β‐amyrin (0.862 Å) and adipedatol BChE (0.752 Å) systems shows that the binding of the secondary minimised the fluctuation around the amino acid residue with enhanced stability compared to the reference compounds decamethonium. This trend was also observed around other catalytic residues (Trp231 and Tyr322) (Figure 9).

The stability of the systems was further investigated by comparing the degree of folding of the enzyme using the RoG parameter. As observed from the RoG plots, the systems were all equilibrated before the end of 10 ns and maintained a stable progression with minimal fluctuations throughout the simulation with a notable convergence at the end of the 100 ns (Figure 8b). Unlike the trend that was observed with the RMSD and RMSF plots, the unbound enzyme and the complex systems presented close progression, as evident from the close mean RoG values, with slightly reduced RoG values in ligand‐bound complexes compared to the apo structure, reflecting the stabilising and compacting effects of ligand interactions. It is interesting to note that adipedatol also showed notable stabilisation across all metrics (RMSD, RMSF and RoG), indicating that it has a considerable impact on the structural dynamics of the protein. The relative similarity in RoG values across most ligand‐bound states highlights the conserved overall shape of the protein, suggesting that ligand binding generally enhances compactness without drastically altering the global structure. The folding pattern of the enzyme was examined using the SASA parameter. The seven systems were equilibrated before 5 ns with a consistent progression throughout the period of the simulation (Figure 8c). The bound systems presented lower mean SASA values when compared to the unbound enzyme. Even though both RoG and SASA thermodynamic parameters are used to measure the compactness of a protein, the result from both parameters slightly differs, with SASA indicating an overall increase in the folded structure of the enzyme upon the binding of the ligands as measured from the amount of surface accessible to water, while RoG did not indicate an increase in the compactness upon the binding of the ligands. The number of hydrogen bonds in the overall protein structure is another indication of the structural stability of the protein. Figure 8d shows the plots of the mean number of hydrogen bonds of the systems across the trajectory. The unbound structure (6EP4_Apo) exhibited a mean H‐bond count of 123.91 ± 8.65, representing the baseline level of hydrogen bonding when ligands are not present. Binding of ligands generally results in a slight reduction in the number of hydrogen bonds, with values ranging from 119.56 to 125.86. 6EP4_DME, and 6EP4_β‐amyrin exhibited similar H‐bond counts (122.36 ± 9.47 and 122.39 ± 9.29, respectively), demonstrating a minor decrease in hydrogen bonding in contrast to the unbound state. 6EP4_TbG (119.56 ± 9.33) and 6EP4_tiliroside (119.96 ± 9.33) had close H‐bond counts, indicating the lowest level of hydrogen bonding among the tested complexes. The 6EP4_DHGB (125.34 ± 9.18) and 6EP4_adipedatol (125.86 ± 8.41) systems showed a slight increase in H‐bond count, implying the strongest stabilising effect among all ligands through enhanced hydrogen bonding. With the highest H‐bond count, adipedatol stands out and demonstrates its potent stabilising effect across various parameters.

### 3.10. Per‐Residue Decomposition of Binding Energy of the Top Five SMs Against the Binding Site of Human BChE

**FIGURE 10:**
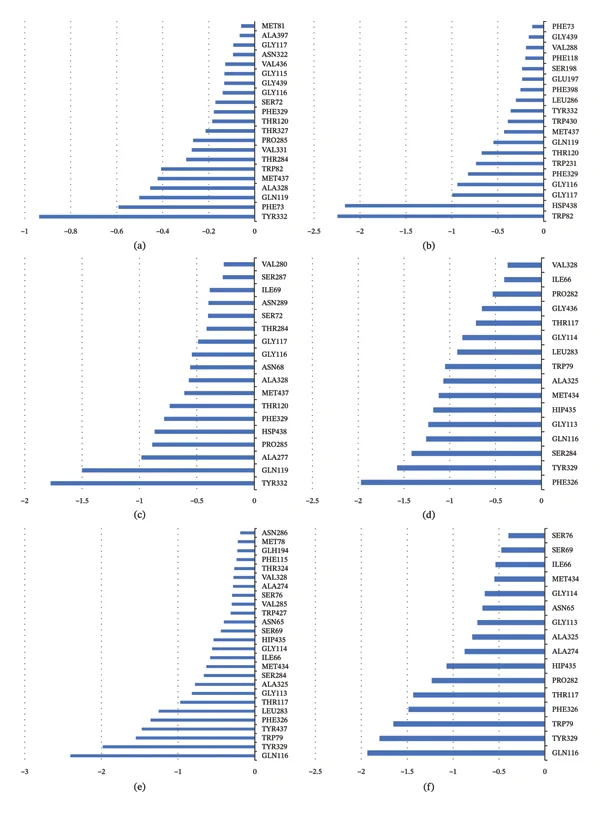
Molecular Mechanics Generalized Born Surface Area (MM‐GBSA) plot of the contributing amino acid residues of BChE to the total binding free energy: (a) decamethonium, (b) 9 (11)‐dehydroergosterol benzoate (DHGB), (c) tiliroside, (d) lupenone, (e) β‐amyrin and (f) adipedatol.

The contributing amino acid to the total binding energy was analysed using the decomposition analysis and is presented in Figure 10. Decamethonium contributed −7.453 to the total BFE, while the remaining were contributed by Tyr332 (−0.936 kcal/mol), Phe73 (−0.592 kcal/mol), Gln119 (−0.501 kcal/mol), Ala328 (−0.454 kcal/mol), Met437 (−0.422 kcal/mol), Trp82 (−0.407 kcal/mol), Thr284 (−0.298 kcal/mol) and Val331 (−0.273 kcal/mol) (Figure 10a). 9 (11)‐Dehydroergosterol benzoate contributed −11.94 kcal/mol to the total BEF. The interaction was stabilised by van der Waals energy (−19.40 kcal/mol) but had relatively low contributions from electrostatic and solvation energies. The amino acids that contributed to the binding of 9 (11)‐dehydroergosterol benzoate include Tyr332 (−1.17 kcal/mol), Gln119 (−1.50 kcal/mol), Gly116 (−0.54 kcal/mol), Thr120 (−0.73 kcal/mol), Ala (−0.98 kcal/mol), Pro285 (−0.88 kcal/mol), Phe329 (−0.78 kcal/mol) and Hsp438 (−0.86 kcal/mol). The analysis further revealed that in order to stabilise the protein–ligand complex through hydrophobic interactions, hydrophobic residues, such as TRP82, TRP231 and PHE329, contributed negatively to van der Waals and nonpolar solvation energies (Figure 10b).

Tiliroside contributed −19.1707 to the total BFE. Of this, van der Waals interactions (−25.84 kcal/mol) and nonpolar solvation (−4.96 kcal/mol) were the major contributors. The major contributing aminos include Ile69 (−0.77 kcal/mol), Trp82 (−1.45 kcal/mol), Gly116 (−1.00 kcal/mol), Gln119 (−0.96 kcal/mol), Glu197 (−0.66 kcal/mol), Ser119 (−1.07 kcal/mol), Trp231 (−2.17 kcal/mol), Pro285 (−1.17 kcal/mol), Leu285 (−1.13 kcal/mol), Leu286 (−1.51 kcal/mol), Phe329 (−2.48 kcal/mol), Tyr332 (−1.68 kcal/mol) and Hsp438 (−1.78 kcal/mol) (Figure 10). Hsp438 and Gln119 show larger contributions to the total binding energy, especially through van der Waals interactions. Tyr332 and Pro285 also contribute significantly, with a mix of polar and nonpolar interactions. With some depending more on polar solvation and others on van der Waals forces, other residues, such as PHE329, GLN119 and THR120, exhibit moderate contributions to the total binding energy (Figure 10c).

For the 6EP4_lupenone system, lupenone contributed (−32.52 kcal/mol) and was driven majorly by strong van der Waals (−28.04 kcal/mol) and nonpolar solvation (−4.15 kcal/mol). The contributing amino acids to the total BFE of the 6EP4_lupenone system were Phe326 (−2.74 kcal/mol), Tyr329 (−1.57 kcal/mol), Ser284 (−1.42 kcal/mol), Gln116 (−1.26 kcal/mol), Gly113 (−1.23 kcal/mol), Hip435 (−1.18 kcal/mol), Met434 (−1.12 kcal/mol), Ala325 (−1.07 kcal/mol), and Trp79 (−1.05 kcal/mol). Residues, such as Phe326, Tyr329 and Ser284, had more moderate contributions, majorly driven by polar solvation and hydrophobic effects, while Gly113, Gln116 and Leu283 presented modest contributions to the binding, primarily due to van der Waals and polar solvation effects (Figure 10d).

For the 6EP4_β‐amyrin system, β‐amyrin contributed (−31.46 kcal/mol) and was driven majorly by strong van der Waals (−27.25 kcal/mol) and nonpolar solvation (−4.30 kcal/mol). The remainder of the BFE was contributed by Gln116 (−2.40 kcal/mol), Tyr329 (−1.98), Trp79 (−1.55 kcal/mol), Tyr437 (−1.47 kcal/mol), Phe326 (−1.36 kcal/mol), Leu283 (−1.25 kcal/mol), Thr117 (−0.97 kcal/mol), Gly113 (−0.82 kcal/mol) and Ala325 (−0.78 kcal/mol). TYR329, TRP79 and TYR437 show the largest contributions to the total energy, with polar solvation being a major positive contributor for some. The other residues contribute weakly to the total energy, with hydrophobic interactions and polar solvation being the primary drivers (Figure 10e).

Adipedatol contributed (−30.75) to the stabilisation of the 6EP4_adipedatol across the trajectories with significant involvement in binding due to hydrophobic and electrostatic interactions. The major contributing amino acids include Gln116 (−1.93 kcal/mol), Tyr329 (−1.80 kcal/mol), Trp79 (−1.65 kcal/mol), Phe326 (−1.49 kcal/mol), Thr117 (−1.43 kcal/mol), Pro282 (−1.23 kcal/mol), Hip435 (−1.07 kcal/mol), Ala274 (−0.87 kcal/mol) and Ala325 (−0.79 kcal/mol). The strongest contributions from Trp79, Gln116 and Tyr329 were majorly driven by hydrophobic interactions and polar solvation. Gly113 and Met434 show moderate contributions to binding with weak interactions across the various energy components. Phe326 and Thr117 exhibit lower stabilising effects, highlighting the variability in residue contributions within the binding pocket (Figure 10f).

## 4. Discussion

In the present study, a ML model was employed for predicting the inhibitory activity of SMs from Nigerian medicinal plants with reported neuroprotective properties against BChE. Molecular modelling studies were further used for predicting the interactions of the ML‐predicted SMs with the human BChE. Although there are few cholinesterase models, none have been used to explore the rich, biodiverse flora of the Nigerian region. Also, there have been previous similar models tailored towards monoamine oxidase inhibition [49, 50] and some BChE 3D‐QSAR studies [50, 51], the present model demonstrated notable improvements in functionality and versatility with enhancement that accommodates larger datasets through a user‐friendly interface that supports CSV file uploads. This feature allows for more extensive data analysis in a single batch, thereby improving the efficiency and scalability of the predictive process [52, 53]. The RF regressor for BChE inhibition shows the extent to which Bayesian optimisation may be used with reliable interpretability frameworks in QSAR modelling. The final model achieved a predictive accuracy that aligns with the inherent experimental variability observed in biochemical assays [54]. Optuna’s Stage 2 hyperparameter optimisation implementation was crucial for enhancing the model’s architecture. The model was able to reach a performance plateau that maximised generalisation while constraining overfitting by implementing a TPE sampler, which investigated the search space more effectively than conventional grid search techniques [55]. The final model showed excellent stability across fivefold cross‐validation, demonstrating that the assigned parameters—n_estimators and max_features, in particular—effectively balanced the bias‐variance trade‐off present in ensemble learning. The use of SHapley Additive exPlanations (SHAP) to go beyond ‘black‐box’ predictions was a crucial component of this investigation. The fragment analysis highlighted a number of structural motifs that closely correspond to the anticipated inhibition of BChE. In particular, amine or amide groups that may establish hydrogen bonds and ionic interactions with the catalytic triad and peripheral anionic site of BuChE are represented by C–N–C linkages (MACCS_bit_82) [56]. This is chemically consistent with the BuChE active site’s known design, which requires a basic nitrogen core to enable cation‐π π interactions with the conserved Trp82 residue in the choline‐binding pocket [57]. By providing aromatic moieties that are capable of π–π stack with active site residues including Trp82 and Tyr332, benzyl or phenyl‐alkyl substituents (Morgan_1357) stabilise ligand binding [58]. Hydrogen bonding and the modulation of lipophilicity are further supported by the methoxy/ether groups (Morgan_667) and carbonyl/amide functionalities (Morgan_1048). Van der Waals contacts are contributed by hydrophobic substituents, such as isopropyl/branched alkyl groups (Morgan_197), while tertiary amines (Morgan_592) are typical characteristics of cholinesterase inhibitors, which produce electrostatic interactions with the catalytic anionic site. Other motifs include heterocyclic nitrogen pieces (Morgan_304), which are recurrent scaffolds, such as pyridine, piperidine or indole, and substituted aromatics (Morgan_1028), which offer electronic and steric diversity affecting binding orientation. While aliphatic linkers (Morgan_1758) provide flexible pharmacophore spot inside the expanded BuChE gorge, halogenated aromatics (Morgan_555) improved lipophilicity and may add halogen bonding interactions. The analysis strengthens confidence in the prediction model’s capacity to direct phytochemical‐based drug discovery by confirming that it captures chemically significant factors of BuChE inhibition. The scaffold‐based partitioning strategy provides additional proof of the model’s robustness. The scaffold split guarantees that the model is evaluated on chemically separate space, even though a lower Test RMSE compared to Training RMSE was noted, a result mostly related to the adoption of cost‐sensitive sample weighting. Furthermore, the Applicability Domain filter is essential, as evidenced by the performance decline seen for compounds ‘Outside AD’. QSAR models are only reliable when the query compounds and the training data have an adequate structural link, as has been demonstrated in the literature [59].

The preliminary exploratory analysis of the bioactivity dataset offered valuable insights into the IC_50_ bioactivity profile within the chemical space of BChE antagonists. The IC_50_ values were transformed to a logarithmic scale (pIC_50_) to ensure a more consistent and statistically controllable depiction of compound potency. Higher potency was specified by a higher pIC_50_ value. A lower pIC_50_ value, however, signifies a decreased potency, meaning that a larger concentration of the compound may be required to get the same therapeutic effect [60]. A compound was regarded as active if its IC_50_ was less than 10,000 nM, which translates to a pIC50 > 5 [61]. In accordance with the reported value, compounds with pIC_50_ values between 5 and 6 were regarded as moderately active, while compounds with pIC_50_ values higher than 6 are referred to as active [62, 63].

The analysis of the pIC_50_ distribution demonstrated a wide chemical space with a core tendency towards moderate BChE inhibition. The pIC_50_ values, for compounds predicted to be potent, ranged from 6.0 to 8.8 for active molecules, contrasting with values < 5 for inactive compounds. This difference highlights the potency of the active compounds as effective inhibitors. The obvious separation between the pIC_50_ values of inactive and active compounds reveals the robustness of dataset used to build and test the model and suggests that the observed biological activity is well‐defined within the ranges [64]. Furthermore, the active compounds presented a Log *p* values between 4.0 and 6.5, in contrast to inactive compounds with values from 3.1 to 5.2. The higher Log *p* values for active compounds imply an optimal hydrophobicity that may enhance their biological membranes crossing ability to interact with the target enzyme [65]. This observation agrees with the understanding that compounds with moderate to high log *p* values are usually effective in biological systems as a result of their enhanced membrane permeability [66].

Furthermore, to reduce the possibility of drug failures that often occur in the later stages of drug development, Lipinski’s ‘Rule of Five’ for orally administrated drugs was integrated into the model [67, 68]. The chemical space investigation showed that most active compounds possessed favourable drug‐like properties. This finding is important as it reveals that the active compounds meet the basic criteria for oral bioavailability in addition to their potential as bioactive molecules. The adherence to Lipinski’s rule highlights the possibility that these compounds are viable candidates for further drug development and optimisation [69].

The ‘Rule of Five’ for drug‐likeness is supported by the median and range of the hydrogen bond acceptor and donor, and molecular weight values for each bioactivity class. These bonds contribute to the stability and specificity of drug‐target complexes, enhancing selectivity and binding affinity [70]. Stable interactions with biological targets often occur in compounds with the right amount of hydrogen bond donors [70]. The hydrogen bond acceptors are central in drug interactions as they enhance the formation of hydrogen bonds with hydrogen bond donors in biological targets [70]. These interactions offer specificity and stability that enhances drug‐target binding, an essential property for therapeutic efficacy [71]. Also, the stabilisation of protein–ligand complex and molecular recognition relies on hydrogen bond acceptors, which are often electronegative atoms, such as oxygen or nitrogen. This property affects the solubility, permeability and general pharmacokinetics of pharmacological compounds, which affects their bioavailability and efficacy [72]. Especially in the active class, the existence of outliers with high HBA values suggests that certain bioactive chemicals may contain a significant number of hydrogen bond acceptors, maybe because of the peculiar binding interactions that BChE may need.

Furthermore, the graphical bar plots show a significantly higher proportion of active compounds compared to inactive ones. This observation agrees with the hypothesis that compounds demonstrating higher bioactivity are usually prevalent in the dataset, potentially reflecting the selection bias inherent in the dataset or the relative scarcity of inactive compounds [73, 74].

Molecular weight is an important factor in drug interactions as it influences a drug’s absorption, distribution, metabolism and excretion (ADME) properties. Compounds with lower molecular weights generally exhibit better permeability and absorption, as they can more easily traverse biological membranes. Furthermore, solubility is influenced by molecular weight; smaller molecules are frequently more water‐soluble, increasing their bioavailability [75].

In this study, the molecular weight distributions revealed that active compounds exhibited a broader range of 380 to 550 Da compared to 300 to 480 Da for inactive compounds. The active compounds presented a higher median MW value when compared to the inactive bioactivity classes. This implies that bioactive compounds may tend to be larger than the inactive compounds, possessing more complex and elaborate structures. This intricacy most likely results from the requirement for molecular characteristics and functional groups that enable particular interactions with biological targets, such as binding sites in proteins or enzymes. Strong and specific drug‐target binding requires a variety of contact locations, such as hydrophobic areas, hydrogen bond donors and hydrogen bond acceptors, which larger molecules may offer [75]. However, these larger structures must strike a balance, as excessive molecular weight can hinder drug permeability and solubility [75].

A rigorous and systematic approach was employed to enhance the accuracy and reliability of the ML model. By identifying and eliminating outliers via applicability domain analysis, the model’s performance and generalisability are enhanced, and anomalous data points are prevented from negatively affecting it [76]. The use of PCA to identify 25 molecules as outliers emphasised the importance of dimensionality reduction techniques in effectively managing complex and high‐dimensional datasets. The optimised RF‐based PubChem fingerprint model exhibited strong predictive performance, as reflected by a high correlation coefficient of 0.8981, which implies a robust linear relationship between predicted and actual values [77]. The RMSEs of 0.7032 and 1.1900 for the training and test sets, respectively [78], along with the MAEs of 0.4556 and 0.8216, reveal that the model exhibits consistent predictive accuracy across both datasets [79]. While the performance on the test set suggests good external validation, the training set’s comparatively lower RMSE and MAE suggest that the model was well‐calibrated without overfitting [80]. Furthermore, the structural analysis of top bioactive molecules—sabinene, limonene and caryophyllene oxide—showed promising results compared to the FDA‐approved cholinesterase inhibitor donepezil. Experimental pIC_50_ values for these molecules were 6.105, 5.556 and 6.161, respectively, while donepezil had a PIC_50_ of 5.7. The RF model predicted pIC_50_ values of 6.1681, 5.7844 and 6.162 for these compounds, and 5.63 for donepezil. These predictions closely align with the experimental values, demonstrating the model’s accuracy and reliability in predicting bioactivity [81].

Creating a robust and comprehensive database is crucial for advancing the discovery of new therapeutics, especially in the context of neurodegenerative diseases, such as AD, to facilitate the exploration of cholinesterase inhibitors from Nigerian medicinal plants. The diversity of medicinal plants with documented anticholinergic properties from the Nigerian flora is revealed by the identified species from 26 distinct families, which supports the idea that neuroprotective compounds are extensively spread throughout the plant kingdom [82]. Among the families, Fabaceae presented the highest number of species with neuroprotective properties followed by Euphorbiaceae. With more than 19,000 species spread across roughly 750 genera, the Fabaceae family—also called Leguminosae—is one of the biggest and most varied groups of flowering plants. Many plants in the Fabaceae family are used in traditional West African and Nigerian medicine to treat age‐related cognitive problems, such as epilepsy, sleeplessness and amnesia [83]. Their pharmacological potential is supported by their historical use, which also makes them attractive candidates for bioprospecting and ML‐assisted neurotherapeutic screening [84]. The data mining of the SMs identified from these plants revealed terpenes, flavonoids and steroids as the most dominant class of SMs. These classes of compounds have been reported to have neuroprotective properties [85–87]. The screening of the compiled SMs afforded 827 that were predicted to be active with pIC50 values ranging from 6.0 to 8.8 highlights the promising prospects of Nigerian medicinal plants as sources of novel cholinesterase inhibitors and suggests that the predicted active compounds could lead to the development of invaluable new therapeutic agents that can be readily available in African countries.

Molecular docking is a crucial step following ML prediction of active compounds, as it provides detailed insights into the interaction between the predicted compounds and their target [88]. The predicted active SMs (827) were thus investigated using molecular docking analysis. The high docking scores of the SMs further confirmed the ability of the model to selectively predict potential inhibitors of BChE. The top 15 SMs with docking scores between 12.0 and −10.3 kcal/mol and lower than donepezil (−8.5 kcal/mol) provided valuable insights into their potential as inhibitors of BChE [89, 90]. From these, the top 5 SMs (tiliroside, lupenone, β‐amyrin, 9 (11)‐dehydroergosterol benzoate and adipedatol) were selected. These leads were identified from Croton gratissimus [91], *Peltophorum pterocarpum* [92, 93], *Bryophyllum pinnatum* [94, 95], *Daedalea elegans* [96, 97] and Alchornea laxiflora [98, 99], respectively. Interestingly, two of the top docked SMs were sourced from plants belonging to the Fabaceae and Euphorbiaceae families.

In addition to the Lipinski filter implemented in the model, the top five SMs identified from docking analysis were further evaluated for ADME, toxicity properties and synthetic feasibility in an effort to reduce the chance of expensive failures that frequently happen at the later stages of drug development procedures [100]. The top five metabolites except tiliroside were likely to be orally bioavailable in its current form and may require, while tiliroside requires optimisation via structural modification to enhance its pharmacokinetic properties [101]. The triterpenoid metabolites, such as β‐amyrin and lupenone, exhibit high MLogP values (> 6), indicating strong hydrophobic character, even though they were compliant with Lipinski’s rule. This property is often associated with poor aqueous solubility, limited dissolution rate and potential bioaccumulation. Therefore, despite their compliance with the Lipinski rules, β‐amyrin and lupenone may be further optimised to reduce lipophilicity and improve solubility [102].

The top five SMs presented high intestinal absorption, which indicates the likelihood that they will be absorbed in the human gut [103]. All the top five SMs were not substrate of P‐glycoprotein (P‐gp). Since the compounds were not a P‐gp substrates, they are less likely to be actively expelled from cells, which may enhance its intracellular concentration and overall bioavailability [104]. Interestingly, all the top five SMs presented high probability of crossing the BBB, which is an important property for neurotherapeutic drug candidate. It implies that these metabolites have the ability to efficiently reach the central nervous system (CNS), allowing for the targeted therapy of neurological conditions, such as AD [105]. Since the barrier’s restrictive nature prevents several drug candidates from treating brain‐related illnesses, these leads’ capacity to permeate the BBB increases their therapeutic potential [106]. Additionally, all the top five SMs were not substrates and inhibitors of most of the cytochrome P450 systems implying that they may bypass metabolism by these enzymes, potentially leading to longer systemic circulation and sustained therapeutic effects. The lack of inhibitory activity also suggests that these SMs are unlikely to disrupt the metabolism of other medications, which lowers the possibility of drug–drug interactions, a typical worry in polypharmacy [107]. The fact that none of the leads are hERG blockers suggests that they are less likely to induce cardiotoxic effects, making them safer candidates for drug development. During clinical trials or postmarket surveillance, this feature lowers the likelihood of adverse cardiac events, which is a frequent cause of medication withdrawal [108]. The absence of predicted human hepatotoxicity for all the top SMs is a highly favourable outcome for their safety and therapeutic potential. None [109] of the hepatotoxic compounds are less likely to harm the liver, which lowers the possibility of side effects, such as increased liver enzymes, jaundice or liver failure. The leads exhibited moderate synthetic accessibility, suggesting that they may not be challenging to synthesise due to factors, such as structural complexity, the presence of rare functional groups or the need for intricate reaction pathways, which often impact the drug discovery and development process [110].

Although the docking analysis provides valuable insight into the possible binding mechanisms of the SMs, this analysis uses a single, inflexible protein structure to investigate the ligand binding potential while ignoring the target binding site’s conformational plasticity limiting the results. Complementary thermodynamic BFE provides a more precise assessment of the thermodynamic stability and binding affinity of ligand–protein complexes [111], while accounting for solvation effects, flexibility and the entropy of the ligand and receptor. BFE are therefore frequently employed to validate the molecular docking predictions. [112]. The result from the BFE analysis corroborated the docking result, with the lead SMs presenting higher negative BEF than the reference compound. The energetics of the lead SM‐BChE systems, especially the top three leads (lupenone, β‐amyrin and adipedatol), revealed a balance of high van der Waals interactions, moderate electrostatic interactions and moderate nonpolar interaction compared to the balance of moderate van der Waals interactions, low electrostatic interactions and nonpolar interaction for the decamethonium‐bound system. The higher van der Waals interactions indicate stronger and more stable hydrophobic contacts, which are essential for efficient binding and activity at the target site [111]. Additionally, the moderate electrostatic and nonpolar interactions in the leads imply a balanced and optimised binding mode that could enhance specificity and reduce off‐target effects [113]. These results demonstrate the lead compounds’ enhancing binding strategy compared to the decamethonium thereby positioning them as promising candidates for cholinesterase inhibition. Also, the finding from this analysis supports a structure‐based design approach, emphasising the optimisation of van der Waals contacts, preserving modest electrostatic contributions balanced for specificity and leveraging on the core scaffolds of lupenone, β‐amyrin and adipedatol for analogue development.

In addition to the BFE analysis, thermostability analysis of the resulting complexes of the leads is crucial. While the former measures the strength and favourability of interactions, the latter examines the structural integrity and resilience of the ligand–target complex under varying thermodynamic conditions. This is crucial because, at physiological or high temperatures, compounds with favourable BFE may fail because the complex becomes unstable. Several thermodynamic parameters were employed to measure the stability of the complexes over time. For example, the RMSD measures the average structural differences between the positions of atoms in a simulated trajectory and a reference structure, thereby indicating the overall stability and conformational changes during the simulation [114]. The SM‐bound system, especially the top three SMs (lupenone, β‐amyrin and adipedatol), presented lower RMSD values compared to the unbound BChE system, thereby suggesting structural stability and minimal deviation from the native conformation of the protein [115]. This implies that despite the high binding affinity to the binding site, the top leads did not destabilise the protein’s three‐dimensional structure, preserving its functional integrity, probably from a highly specific interaction with the protein in a controlled manner that enhanced their binding affinity while reducing the risk of off‐target effects or undesired structural alterations [116]. This trend was also observed from the binding of leads coumarins with high binding free energies [117]. To corroborate the stability from the RMSD assessment, the RMSF parameters that assess the degree of flexibility for specific protein regions or residues of the protein [118] were investigated. The results reveal that the low RMSF values for the global structure and key catalytic residues, such as Tyr82, in the lupenone‐, β‐amyrin‐ and adipedatol‐bound systems highlight a significant reduction in the flexibility of the protein upon ligand binding. This implies that SMs stabilised the protein structure, particularly around catalytically important regions, which is crucial for maintaining enzymatic activity and function [116].

Comparatively, the complexes of the three top leads with enhanced stability presented the highest negative BFE. This relationship reflects the interplay between structural stability, flexibility and high binding affinity in protein–ligand complexes. The low reduced deviations in the overall structure of the protein–ligand complex and stabilisation of the binding regions may have optimised the interaction of the leads by minimising unwanted movements, thereby forming strong and specific interactions, which may have contributed to high negative BFE [119]. The observed stabilisation of the BChE structure upon the binding of the leads aligns with the binding of the most potent pregnanes in previous studies [120, 121]. In one of these studies, enhanced stability and higher negative BFE of the lead pregnane (iloneoside) corroborated with the high inhibition of BChE in vitro [120].

The RoG and SASA thermodynamic parameters were also employed to assess the compactness of the bound system upon the binding of the leads. While the RoG measures the distribution of atoms around the centre of mass of the system [115], the SASA parameter reflects the surface area of the molecule exposed to the solvent [122]. The binding of the top three leads in this study resulted in reduced RoG and SASA. The reduced RoG indicates that the protein–SM complex becomes more compact upon binding. This structural compactness is beneficial as it indicates a stable conformation with minimal structural fluctuations, reinforcing the overall stability and minimal distortion in alignment with the low RMSD values. In the same vein, the development of stable protein–ligand connections correlates with the reduction of hydrophobic residues’ accessibility to the solvent, as indicated by the decrease in SASA [122]. The enhanced overall stability upon the binding of the lead SMs may have resulted from the increased mean number of hydrogen bonds in the lead SM–bound complexes. The enhanced hydrogen bonds in the top three lead complexes of BChE were also observed in similar studies [123, 124]. The increased number of hydrogen bonds may have further contributed to the observed stability by reinforcing intermolecular interactions, reducing the conformational freedom, making the complex more tightly packed and potentially shielding certain areas from the solvent [125].

Furthermore, to understand the contribution of individual amino acid residues to the binding process between a lead SMs and BChE, per‐residue decomposition of total BFE was performed, thus serving as a complementary analysis to the global BFE calculation. The observation of the contributing residue revealed that the top three leads maintain contact with the key catalytic residues (Try82 Trp231 and Phe329) in the A‐site and Tyr332 of the P‐site, and Asp70, which is located near the flip of the BChE active site gorge [36]. Asp70 and Trp82 of the cysteine Ω‐loop are known to form a direct linkage between the choline‐binding pocket in the A‐site and P‐site [126]. Strong interactions between these catalytic residues and compounds, such as decamethonium, have been reported to inhibit the enzymes’ catalytic function.

Although this study offers important conclusion, it is important to acknowledge its several limitations. First, the finding is completely based on computational methods; hence, experimental validation is needed. Second, the intrinsic limits of simulation time scales, force fields and the scoring functions of the molecular docking algorithm used could limit the precision of anticipated binding affinities and stability profiles. Also, the authors acknowledge limitations due to force field and solvation model used in the study. Lastly, because pharmacokinetic and toxicity evaluation were entirely in silico, safety must be confirmed empirically. Consequently, in vitro and in vivo investigations are needed to substantiate the therapeutic effectiveness of the identified leads.

## 5. Conclusion

In this multistep computational study, an integrated ML and molecular modelling investigation of SMs from Nigerian medicinal plants with reported cholinesterase inhibitory activity against BChE revealed five leads (tiliroside, lupenone, β‐amyrin, 9 (11)‐dehydroergosterol benzoate and adipedatol) with predicted high bioactivity and complementary high binding affinity for the BChE. These five leads demonstrated higher negative thermodynamic BFE against the BChE target when compared to the reference compound decamethonium, signifying stronger and more thermodynamically favourable interactions. The lead SM‐BChE systems were stabilised by strong van der Waals, moderate electrostatic and nonpolar interactions, compared to moderate van der Waals, low electrostatic and nonpolar interactions in the decamethonium‐bound system. This diversified interaction mechanism might contribute to improved binding resilience and activity in complex biological environments. Among the five leads, β‐amyrin, lupenone and adipedatol were the most promising SMs. These leads promise superior inhibitory potential of BChE that may result in a more effective modulation of the enzyme’s activity compared to the reference standard. The favourable ADMET properties, coupled with the promising safety profiles, strongly support the potential of these leads as suitable candidates. Furthermore, the binding of lead SMs optimised the structural and interaction profiles that preserved the structural integrity of the enzyme and aligned well with favourable drug‐like characteristics. Overall, the findings from this study position the leads as putative candidates for therapeutic applications against BChE. Although the study presents strong computational evidence that supports the inhibitory potential of the lead SMS against BChE, in vitro and in vivo studies will be the focus of future research to validate their neurotherapeutic potential. Also, lead optimisation by SAR research may enhance the selectivity of binding and of these leads. Modification of the triterpenoid scaffolds that is common to the top three leads may improve potency and reduce potential off‐target effects.

**Nomenclature**
BChE: Butyrylcholinesterase
AD: Alzheimer’s diseaseApplicability domain (APD)
APD: Applicability domain
ML: Machine learning
SMs: Secondary metabolites
Aβ: β‐Amyloid
AChE: Acetylcholinesterase
PD: Parkinson’s disease
GUI: Graphical user interface
SARs: Structure–activity relationships
SMILES: Simplified Molecular Input Line Entry System
WoS: Web of Science
CSV: Comma‐separated value
VS code: Visual Studio Code
SDF: Structured data format
hAChE: Human butyrylcholinesterase
LINCsS: LINear Constraint Solver
MAEs: Mean absolute errors
RSE: Relative squared error
DEM: Decamethonium
DHGB: 9 (11)‐Dehydroergosterol benzoate
Δ_VDWAALS_
: Change in van der Waals energy
Δ_EEL_
: Change in electrostatic energy
Δ_EGB_
: Change in electrostatic potential energy
Δ_ESUR_
: Change in surface energy
Δ_GGAS_
: Change in the Gibbs free energy of the gas phase
ΔG_SOLV_
: Change in Gibbs free energy of solvation
Δ_TOTAL_
: Total energy change

## Author Contributions

Conceptualisation: Gideon Ampoma Gyebi; methodology and software: Gideon Ampoma Gyebi and Onomeyimi O. Onesi; formal analysis: Onomeyimi O. Onesi and Gideon Ampoma Gyebi; writing–original draft preparation: Onomeyimi O. Onesi and Gideon Ampoma Gyebi; writing–review and editing: Gideon Ampoma Gyebi and Saheed Sabiu; and supervision: Saheed Sabiu.

## Funding

This work did not receive any funding from external sources.

## Disclosure

All authors have read and agreed to the published version of the manuscript.

## Conflicts of Interest

The authors declare no conflicts of interest.

## Supporting Information

Additional supporting information can be found online in the Supporting Information section.

## Supporting information


**Supporting Information** Supporting files: Database of plants. ML_Tranining data. ML_Testing data. Table S1: Presents the list of Nigerian plants with report anticholinergic activities.

## Data Availability

All data are provided in the article and its Supporting Information.
