# Supplementary material for: Machine Learning–Driven Bioprospecting of Cholinergic Medicinal Plants for Putative Leads Against Butyrylcholinesterase Towards Alzheimer’s Care
Source: Biochem Res Int. 2026 Aug 31;2026:4503245. doi: 10.1155/bri/4503245 (PMC13528771; doi:10.1155/bri/4503245)
Supplement: Supplementary file 1 [file BRI-2026-4503245-s001.docx]

**Supplementary data**

**Table S1:** List of Nigerian plants with report anticholinergic activities

| **S/N** | **Species** | **Family** | **Part** | **Common Name** | **Region** | **Class of Compounds** | **Reference** |
| --- | --- | --- | --- | --- | --- | --- | --- |
| 1 | Abrus *precatorius L* | Fabaceae | Seeds | Ojuologbo | Western Nigeria | Alkaloids, Flavonoids, Tannnins, Terpines | (Garaniya et al., 2018), (Deepa & Farshana, 2021), (Gawade & Farooqui, 2021) |
| 2 | Acacia *nilotica* | Fabaceae | Bark | Bagarwa | Northern Nigeria | Phenolic compounds, Terpenoids | (Rehman et al., 2022), (Abubakar et al., 2022) |
| 3 | Acanthus *montanus* | Acanthaceae | Leaf  Root | Ogwu ahga | Eastern Nigeria | Sterol derivatives, Phenolic compounds | (Getahun et al., 2023)  (Ou et al., 2018)  (Getahun et al., 2023)  (Okoli et al., 2008) |
| 4 | Achillea *millefolium* | Asteraceae | Leaves | Yarrow | South Africa | Terpenoids | (Smelcerovic et al., 2009), (Raudone et al., 2024) |
| 5 | Adenia *cissampeloides* | Passifloraceae | Stem Bark | Ibo Eze | South Eastern Nigeria | Terpenoids, Esters | (Ogunlesi et al., 2019) |
| 6 | Aframomum *melegueta* | Zingiberaceae | Seed | Atare | Nigeria | Phenols  Terpenoids, Heterocyclic compounds | (Ambali, 2021)  (Asiat et al., 2020) |
| 7 | Albizia *adianthifolia* | Fabaceae | Leaves | Ayunrebonabona | Nigeria | Flavonoid  Terpenoids  Sterols | (Maroyi, 2018), (Mubo & Ibukun, 2015) |
| 8 | Alchornea *cordifolia* | Euphorbiaceae | Bark, Leaves | Christmas bush | Northern Nigeria | Phenolic compounds, Terpenoids  Sterols | (Enyiukwu et al., 2024)  (Essien et al., 2015)  (Boniface et al., 2016)  (Noundou et al., 2016)  (Foko et al., 2023) |
| 9 | Alchornea *laxiflora* | Euphorbiaceae | Leaves | Lowveld bead-string | South Eastern | Flavonoid,  Phenol  Ester | (Jain et al., 2022), (Dzoyem & Eloff, 2015) |
| 10 | *Alcyone laxiflora* | *Euphorbiaceae* | *Leaves* | Pepe, Ijan, Uwenuwen, | Nigeria | Phenol  Flavonoids | (Bafor *et al.*, 2018; Esan *et al.*, 2024) |
| 11 | Allium *cepa* | Amaryllidaceae | Leaves | Alubosaelewe | Nigeria | Flavonoids  Glycosides  Thiols | (Teshika et al., 2018), (Mubo & Ibukun, 2015) |
| 12 | Allium *sativum* | Amaryllidaceae | Bulb | Ayuu | South West Nigeria | Phenols  Esters | (Yunus et al., 2021) (Herrera-Calderon et al., 2021) |
| 13 | Alstonia *boonei* | Apocynaceae | Bark, Leaves | onyema dua | Southern Nigeria | Terpenoids  Fatty acids  Phenolic compounds  Esters | (Okoye et al., 2022), (Babatunde, , 2017), (Oyebode et al., 2019) |
| 14 | Amaranthus *viridis L* | Amaranthaceae | Leaves, Seeds | African spinach | Southern Nigeria | Terpenoids  Phenolic compounds | (Naeem et al., 2022), (Salvamani et al., 2016), (Sunday & Gift, 2021), (Singh & Bency, 2023) |
| 15 | Ammocharis *coranica* | Amaryllidaceae | Bark | Kaffir Lily |  | Triterpenoids  Sesquiterpenes | (Koorbanally et al., 2000), (Masondo et al., 2019) (Elisha, 2011) |
| 16 | Anacardium *occidentale* | Anacardiaceae | Leaf  Seed | Cashew  Kaju  Kahu  Okpokpo | Nigeria | Phenolic compounds  Flavonoids  Stigmasterols | (Salehi et al., 2019), (Thomas et al., 2015) |
| 17 | Andrographis *paniculata* | Acanthaceae | Aeriel Portion | Meje meje | Western Nigeria | Diterpenoids | (Bhatnaga, 2023) |
| 18 | Asparagus *africanus* | Asparagaceae | Root | Shekan bera | Northern Nigeria | Xanthones  Flavonoids  Andrographolides | (El-Ishaq et al., 2019), (Hassan et al., 2008)  (Mfengwana & Mashele, 2020) |
| 19 | Asparagus *densiflorus* | Asparagaceae | Root | Inwele | Southern Nigeria | Flavonoids  Phenolic Content | (Hamdi et al., 2021), (Joshi et al., 2010), (Mfengwana & Mashele, 2020) (Adeniran, 2024) |
| 20 | Asparagus *officinalis L.* | Asparagaceae | Shoots  Leaves | Ehlilaj aswad | Northern Africa | Saponins  Glycosides  Phenylpropene | (Joshi et al., 2010), (Mfengwana & Mashele, 2020) |
| 21 | Azadirachta *indica* | Meliaceae | Leaf | Dongoyaro | Western Nigeria | Triterpenoids  Esters  Saponin | (Raissa et al., 2019), (Hossain et al., 2013), (Oyedemi et al., 2017) |
| 22 | Bacopa *floribunda* | Scrophulariaceae | Leaves | Oniyemiye | Nigeria | Triterpenoids  Lactones | (Adewole et al., 2022),(Mubo & Ibukun, 2015) |
| 23 | Blighia *sapida* | Sapindaceae | Seed, Fruit | Ackee | Nigeria | Flavonoids, Alkanois  Terpenes  Saponins | (Ojo et al., 2018), (Adekola et al., 2020), (Edewor et al., 2016), (Ibraheem et al., 2022), |
| 24 | Blighia *unijugata* | Sapindaceae | Root, bark, leaf | Gwanja-kusa | Northern Nigeria | Terpenoids  Phenolic compounds  Alkylamines | (Dorcas et al., 2017), (Oyedemi et al., 2017) |
| 25 | Boerhavia *diffusa L.* | Nyctaginaceae | Root, Leaves | Etiponla | Western Nigeria | Flavonoids  Terpenoids  Phenolic compounds  Steroids | (Sharma & Veer, 2021), (Jana et al., 2021), (Beegun et al., 2014), (Nayak & Thirunavoukkarasu, 2016) |
| 26 | Bombax *buonopozense* | Malvaceae | Leaves | Eso | Nigeria | Esers  Steroids  Triterpenoids | (Osuntokun et al., 2021), (Mubo & Ibukun, 2015) |
| 27 | ***Bryophyllum pinnatum*** | ***Crassulaceae*** | ***Leaves*** | Abamoda | Nigeria | Terpenes  Flavonoids | (Gaind & Gupta, 1972; Ojo *et al.*, 2018) |
| 28 | Cajanus *cajan (L.)* | Fabaceae | Seeds, Leaves | Pigeon pea | Northern Nigeria | Phenolic  Carboxylic acid derivatives  Isoflavones | (Dinore et al., 2024), (Vasudevan et al., 2023), (Gargi et al., 2022) |
| 29 | Capsicum *annuum* | Solanaceae | Fruit | Tatashe | South West Nigeria | Terpenoids  Esters  Fatty acids | (Wesołowska et al., 2011) |
| 30 | Carica *papaya* | Caricaceae | Leaves | Okwuru-ezi | South Eastern Nigeria | Phenols  Steroids  Terpenes | (Adetuyi et al., 2024), (Al-Seadi et al., 2021), (Adetuyi et al., 2024) |
| 31 | Carthamus *tinctorius* | Asteraceae | Flower | Safflower | South West Nigeria | Flavonoids  Phenols  Glycosides | (S. Wang et al., 2021), (Ingkaninan et al., 2003) |
| 32 | Cassia *sieberiana* | Fabaceae | Leaves, Bark | Margaje Ifo | Western Nigeria, Northern Nigeria | Esters  Flavoinoids  Phenolic Compounds | (Archer et al., 2021), (Macedo et al., 2021), (Akhigbemen et al., 2022), |
| 33 | ***Croton. gratissimus*** | Euphorbiaceae | Leaves | Lavender croton | South East Nigeria | Phenol  Flavonoids | (Ndhlala *et al.*, 2013) |
| 34 | ***Daedalea elegans*** | ***Polyporaceae*** | ***Whole*** | Elegant bracket fungus | Nigeria | Phenol  Flavonoids | (Mazumder & Sarkar, 2023; Mensah-Agyei *et al.*, 2020) |
| 35 | Erythrophleum *africanum* | Fabaceae | Leaves, Bark | Arican rosewood | South-East and North central, Nigeria | Esters  Steroids  Fatty acid derivatives | (Joshua et al., 2023) |
| 36 | Erythrophleum *suaveolens* | Fabaceae | Bark, Seed, Leaves | Bitter kola | South-West Nigeria | Esters | (Mohammed et al., 2014), (Son, 2019), (Dade et al., 2015) |
| 37 | Euphorbia *hirta*. | Euphorbiaceae | Leave, Stems | Asthma plant | Southern Nigeria | Flavonoids  Polyphenols  Triterpenoids  Sterols | (Rautela et al., 2020), (Igwe et al., 2016), (Kain et al., 2022), (De & Sharma, 2023), (Poorna, Anu, & Sathiyajith J N, 2022), (Bakar et al., 2020) |
| 38 | Ficus *lutea* | Moraceae | leaves | Lagos rubbertree | Western Nigeria | Steroids  Esters  Flavonoids | (Elhawary et al., 2018), (Fozeng et al., 2023), (Fozeng et al., 2023), |
| 39 | *Gongronema latifolium Benth* | *Asclepiadaceae* | *Leaves* |  | South West Nigeria | Phenol  Flavonoids  Steroids | (Gyebi *et al.*, 2023; Gyebi *et al.*, 2024) |
| 40 | Mangifera *indica* | Anacardiaceae | Leaves | Mango | Northern Nigeria | Phenols  Terpenoids  Sphingolipids  Phthalates | (Ayoola et al., 2020), (Adeyinka et al., 2022), (Mary Helen et al., 2013), (Vilela et al., 2013), (Mercija & Nevaditha, 2022), (Karumanchi et al., 2016), (Mercija & Nevaditha, 2022) |
| 41 | Moringa *oleifera* | Moringaceae | Leaves | Barambo | Northern Nigeria | Phenol  Flavonoids | (J. Wang et al., 2024), (Abubakar et al., 2022) |
| 42 | Ocimum *gratissimum* | Lamiaceae | Leaves, Flower | Efinrin | Western Nigeria | Terpenoids  Phenols  Esters | (Gyebi et al., 2022) |
| 43 | Peltophorum *pterocarpum* | Fabaceae | Flower | Yellow-flamboyant | Nigeria | Fatty acid nderivatives  Terpenes  *Flavonoids* | (Jash et al., 2014), (Monehin et al., 2024) |
| 44 | ***Peltophorum Pterocarpum*** | ***Fabaceae*** | ***Flowers*** | Yellow flame tree | South West Nigeria | Terpenes  Flavonoids | (Jash *et al.*, 2013; Monehin *et al.*, 2024) |
| 45 | *Piper nigrum* | *Piperaceae* | *Fruit* |  | South Nigeria | Terpenes  Flavonoids | (Ademuyiwa *et al.*, 2023) |
| 46 | *Spondias mombin (Ijebu)* | *Anacardiaceae* |  |  |  |  | (Elufioye *et al.*, 2017; Okereke *et al.*, 2021) |
| 47 | Talinum *triangulare* | Portulacaceae | Leaves | Gbure | Nigeria | Phenols  Polyphenols  Glycosides | (Ikewuchi et al., 2016), (Mubo & Ibukun, 2015) |
| 48 | *Vernonia amygdalina* | *Asteraceae* | *Leaves* |  | South West Nigeria | Terpenes  Flavonoids | (Igbinidu & Nimenibo Uadia, 2019) |
| 49 | ***Waltheria indica*** | Sterculiaceae | Leaves | Ewe-epo | South West Nigeria | **Flavonoids** | (Ayinde *et al.*, 2021) |
| 50 | Zingiber *officinale* | Zingiberaceae | Stems | Ata Ile | South West Nigeria | Phenols  Terpenoids  Cylohexanes | (Momoh & Olaleye, 2022) |

**References**

Abubakar, I. B., Kankara, S. S., Malami, I., Danjuma, J. B., Muhammad, Y. Z., Yahaya, H., Singh, D., Usman, U. J., Ukwuani-Kwaja, A. N., Muhammad, A., Ahmed, S. J., Folami, S. O., Falana, M. B., & Nurudeen, Q. O. (2022). Traditional medicinal plants used for treating emerging and re-emerging viral diseases in northern Nigeria. *European Journal of Integrative Medicine*, *49*, 102094.

Abubakar, I. B., Kankara, S. S., Malami, I., Danjuma, J. B., Muhammad, Y. Z., Yahaya, H., Singh, D., Usman, U. J., Ukwuani-Kwaja, A. N., Muhammad, A., Ahmed, S. J., Folami,

Adekola, M., Areola, J., Oriyomi, O., Apata, J., Apalowo, O., Adesina, A., & Babalola, O. O. (2020). brine shrimps’lethality test of ethanol extract and gas chromatography-mass spectrometry analysis of ethyl acetate fraction of *blighia sapida*. *Asian Journal of Pharmaceutical and Clinical Research*, 208–212

Ademuyiwa, O. H., Fasogbon, B. M., & Adebo, O. A. (2023). The potential role of Piper guineense (black pepper) in managing geriatric brain aging: a review. *Critical reviews in food science and nutrition*, *63*(16), 2840-2850.

Adeniran, O. (2024). Exploring the therapeutic potential of tomato varieties in Alzheimer's disease: A focus on radical scavenging abilities and acetylcholinesterase inhibition. *COAST*, *6*(1), 989-999.

Adetuyi, A. R., Ayenero, M. E., Olaleye, M. T., Akindahunsi, A. A., & Akinmoladun, A. C. (2024). Antioxidant and acetylcholinesterase inhibitory activities, in silico analyses, and anti-Alzheimer’s disease potential of leaf extracts of three Nigerian endemic medicinal plants (Spondias mombin, Carica papaya and Kalanchoe crenata). *Future Journal of Pharmaceutical Sciences*, *10*(1).

Adewole, E., Ogola, E., & David, P. E. (2022). Insecticidal Potentials and Identification of Bioactive Compounds from Extracts of Bacopa Floribunda and LudwigiaDecurrens Plants. *Journal of Agriculture and Crops*, *84*, 345–351Adewole, E., Ogola, E., & David, P. E. (2022). Insecticidal Potentials and Identification of Bioactive Compounds from Extracts of Bacopa Floribunda and LudwigiaDecurrens Plants. *Journal of Agriculture and Crops*, *84*, 345–35.

Adeyinka, O. A., Bankole, I. a. S., & John, R. (2022). GC-MS bioprospecting of phytochemicals in the ethanolic extract of fresh Mangifera indica LEAVES. Journal of Chemical Society of Nigeria, 47(4).

Akhigbemen, A., Okorie, J., Ehiejirikwe, I. C., & Owolabi, O. J. (2022). Bioactive constituents and neuropharmacological evaluation of *Cassia sieberiana*dc (fabaceae) in murine models. [*www.nijophasr.net*](http://www.nijophasr.net).

Al-Seadi, H. L., Sabti, M. Z., & Taain, D. A. (2021). GC-MS Analysis of papaya leaf extract (Carica papaya L.). *IOP Conference Series Earth and Environmental Science*, *910*(1), 012011.

Ambali, O. A. (2021). Bioassay-led isolation of cytotoxic compounds from extracts of aframomum melegueta(roscoe). *ResearchGate*.

Archer, M., Kyene, M. O., Kumadoh, D., Yeboah, G. N., Ayertey, F., Mintah, S. O., Brews-Daniels, H., Agyei, T. A., Appiah, A. A., & Jnr, P. A. (2021). Phytochemical, elemental and functional group analyses of herbal material and extracts of Cassia sieberiana used in herbal drug formulation. *Journal of Medicinal Plants Research*, *5*(10), 490–502.

Asiat, N., Oloruntoyin, A. Y., Ibukun, K. D., Sobiat, S., Bukola, B., Adewunmi, A. M., Abdul-Azeez, B., Titilola, A. A., & Nizam, M. M. (2020). Phytochemical screening and in silico pharmacological profiling of ethanolic extract of Aframomum melegueta for prostate carcinoma. *Journal of Applied Pharmaceutical Science*.

Ayinde, B., Owolabi, J., Uti, I., Ogbeta, P., & Choudhary, M. (2021). Isolation of the antidiarrhoeal tiliroside and its derivative from Waltheria indica leaf extract. *Nigerian Journal of Natural Products and Medicine*, *25*(1), 86-92.

Ayoola, A. A., Ekunseitan, D. A., Muhammad, S. B., Oguntoye, M. A., & Adejola, Y. A. (2020). Phytochemicals analysis and GC-MS determination of ethanolic extracts of Azadirachta indica and Mangifera indica stem bark and their biological potentials. Pacific Journal of Science and Technology, 21(9), 219-229.

Babatunde, O. (2017). GC-MS analysis of leaf, Stem-Bark and root extracts of Alstonia boonei. *African Journal of Pharmacy and Pharmacology*, *11*(46), 577.

Bafor, E. E., Nwogu, J. K., Elvis-Offiah, U. B., Amaechina, F., Ofeimun, J., Ayinde, B., Omoruyi, O., Viegelmann, C., & Edrada-Ebel, R. (2018). Modulation of ex-vivo uterine contraction by the methanol leaf extract of Alchornea laxiflora Benth.(Euphorbiaceae) and preliminary spectrometric identification of associated secondary metabolites. *Journal of Medicinal Plants for Economic Development*, *2*(1), 1-13.

Bakar, F. I. A., Bakar, M. F. A., Abdullah, N., Endrini, S., & Fatmawati, S. (2020). Optimization of Extraction Conditions of Phytochemical Compounds and Anti-Gout Activity of Euphorbia hirta L. (Ara Tanah) Using Response Surface Methodology and Liquid Chromatography-Mass Spectrometry (LC-MS) Analysis. *Evidence-based Complementary and Alternative Medicine*, *2020*(1).

Boniface, P. K., Ferreira, S. B., & Kaiser, C. R. (2016). Recent trends in phytochemistry, ethnobotany and pharmacological significance of Alchornea cordifolia (Schumach. & Thonn.) Muell. Arg. *Journal of Ethnopharmacology*, *191*, 216–244.

Dade, J. M., Kablan, L. A., Okpekon, T. A., Say, M., Yapo, K. D., Komlaga, G., Boti, J. B., Koffi, A. P., Guei, L. E., Djakoure, L. A., & Champy, P. (2015). Cassanediterpenoids from stem bark of Erythrophleumsuaveolens [(Guill. et Perr.), Brenan]. *Phytochemistry Letters*, *12*, 224–231.

Deepa, P., & Farshana, K. L. (2021). In vitro salt tolerance induced secondary metabolites production in Abrus precatorius L. *Current Botany*, 115–120.

Dinore, J. M., Patil, H. S., Farooqui, S. F., Nagare, K. R., Ambhore, J. S., & Farooqui, M. (2024). Exploring Bioactive Compounds in Cajanus cajan (L.) Millsp. Stem and their biological activities: Integration of GC-MS and LC-MS Techniques. *The Journal of Phytopharmacology*, *13*(2), 133–142.

Dorcas, O. M., Usman, Z. F., Oludoyin, A. A., & Clement, O. A. (2017). Essential oil compositions of leaf, stem-bark, stem, root, flower, and fruit with seed of Blighia unijugata Baker (Sapindaceae). *African Journal of Pharmacy and Pharmacology*, *11*(7).

Dzoyem, J. P., & Eloff, J. N. (2015). Anti-inflammatory, anticholinesterase and antioxidant activity of leaf extracts of twelve plants used traditionally to alleviate pain and inflammation in South Africa. *Journal of Ethnopharmacology*, *160*, 194–201.

Edewor, T., Nimotalai, O., & Owa, S. O. (2016). Gas chromatography-mass spectrometric analysis of methanolic leaf extracts of Lannea kerstingii and Nauclea diderrichii, two medicinal plants used for the treatment of gastrointestinal tract infections. *Sian Journal of Pharmaceutical and Clinical Research*, *9*(4), 179–182.

Elhawary, S. S., Younis, I. Y., Bishbishy, M. H. E., & Khattab, A. R. (2018). LC–MS/MS-based chemometric analysis of phytochemical diversity in 13 Ficus spp. (Moraceae): Correlation to their in vitro antimicrobial and in silico quorum sensing inhibitory activities. Industrial Crops and Products, 126, 261–271.

Elisha, I. L. (2011). *Characterization of an acetylcholinesterase inhibitor isolated from Ammocharis coranica (Amaryllidaceae)*. University of Pretoria (South Africa).

El-Ishaq, A., Alshawsh, M. A., Mun, K., & Chik, Z. (2019). Phytochemical screening and anti-implantation activity of Asparagus africanus root extract in female Sprague–Dawley rats. *Revista Brasileira De Farmacognosia*, *29*(5), 621–630.

Elufioye, T. O., Obuotor, E. M., Agbedahunsi, J. M., & Adesanya, S. A. (2017). Anticholinesterase constituents from the leaves of Spondias mombin L.(Anacardiaceae). *Biologics: Targets and Therapy*, 107-114.

Enyiukwu, D. N., Amadioha, A. C., & Bassey, I. N. (2024). Ethnobotany and phytochemical composition of Alchornea cordifolia in Abia State Nigeria. *International Journal of Traditional and Complementary Medicine Research*.

Esan, O. O., Igado, O. O., Femi-Akinlosotu, O. M., Oyagbemi, A. A., Omobowale, T. O., Oladele, O. A., & Nwulia, E. (2024). Alchornea laxiflora (Benth.) Pax & K. Hoffman extract protects against lead-induced neurodegeneration in cockerel chickens. *IBRO Neuroscience Reports*, *17*, 65-72.

Essien, E., Newby, J., Walker, T., Setzer, W., & Ekundayo, O. (2015). Characterization and Antimicrobial Activity of Volatile Constituents from Fresh Fruits of Alchorneacordifolia and Canthium subcordatum. *Medicines*, *3*(1), 1.

Foko, L. P. K., Hawadak, J., Verma, V., Kedi, P. B. E., Moukoko, C. E. E., Kamaraju, R., Pande, V., & Singh, V. (2023). Phytofabrication and characterization of Alchornea cordifolia silver nanoparticles and evaluation of antiplasmodial, hemocompatibility and larvicidal potential. *Frontiers in Bioengineering and Biotechnology*, *11*.

Fozeng, H. D. S., Donfack, A. R. N., Tchuenmogne, M. a. T., Tchegnitegni, B. T., Matchide, M. G. T., Matheuda, E. G., Djoumbissie, R. a. D., Nguekeu, Y. M. M., Ngouela, S. A., Ali, M. S., Awouafack, M. D., & Tene, M. (2023). A new ceramide and other constituents from the fruits of Ficus lutea Vahl (Moraceae) and their chemotaxonomic significance. Biochemical Systematics and Ecology, 106, 104573.

Gaind, K., & Gupta, R. (1972). Alkanes, alkanols, triterpenes and sterols of Kalanchoe pinnata. *Phytochemistry*, *11*(4), 1500-1502.

Garaniya, N., Golakiya, B., Bapodra, A., & Solanki, B. (2018).Phytopharmacological study of methanolic seed extracts of red Abrus precatorius L. [*www.phytojournal.com*](http://www.phytojournal.com).

Gargi, B., Semwal, P., Pasha, S. B. J., Singh, P., Painuli, S., Thapliyal, A., & Cruz-Martins, N. (2022). Revisiting the Nutritional, Chemical and Biological Potential of Cajanus cajan (L.) Millsp. *Molecules*, *27*(20), 6877.

Gawade, B., & Farooqui, M. (2021). Antioxidant Potential and GC-MS Analysis of Abrus precatorius Linn Leaves Ethanol Extract. *Chemical Science International Journal*, 39–46.

Getahun, M., Nesru, Y., Ahmed, M., Satapathy, S., Shenkute, K., Gupta, N., & Naimuddin, M. (2023). Phytochemical Composition, Antioxidant, Antimicrobial, Antibiofilm, and Antiquorum Sensing Potential of Methanol Extract and Essential Oil from Acanthus polystachyus Delile (Acanthaceae). *ACS Omega*, *8*(45), 43024–43036.

Gyebi, G. A., Ejoh, J. C., Ogunyemi, O. M., Ibrahim, A. M., Ibrahim, I. M., Afolabi, S. O., Anyanwu, G. O., Ojo, R. J., Ogunro, O. B., & Alotaibi, B. S. (2023). Probing the multitargeting potential of n-hexane fraction of Gongronema latifolium leaves in neurodegeneration via in vitro, GC–MS and in silico studies. *Future Journal of Pharmaceutical Sciences*, *9*(1), 84.

Gyebi, G. A., Ogunyemi, O. M., Ibrahim, I. M., Afolabi, S. O., Ojo, R. J., Ejike, U. D., & Adebayo, J. O. (2022). Inhibitory potentials of phytocompounds fromOcimum gratissimum against anti-apoptotic BCL-2 proteins associated with cancer: an integrated computational study. Egyptian Journal of Basic and Applied Sciences, 9(1), 588–608.

Gyebi, G. A., Ogunyemi, O. M., Ibrahim, I. M., Ogunro, O. B., Afolabi, S. O., Ojo, R. J., Anyanwu, G. O., El-Saber Batiha, G., & Adebayo, J. O. (2024). Identification of potential inhibitors of cholinergic and β-secretase enzymes from phytochemicals derived from Gongronema latifolium Benth leaf: An integrated computational analysis. *Molecular Diversity*, *28*(3), 1305-1322.

Hamdi, A., Jaramillo-Carmona, S., Rodríguez-Arcos, R., Jiménez-Araujo, A., Lachaal, M., Karray-Bouraoui, N., & Guillén-Bejarano, R. (2021). Phytochemical Characterization and Bioactivity of Asparagus acutifolius: A Focus on Antioxidant, Cytotoxic, Lipase Inhibitory and Antimicrobial Activities. *Molecules*, *26*(11), 3328.

Hassan, H., Ahmadu, A., & Hassan, A. (2008). Analgesic And Anti-Inflammatory Activities Of <i>Asparagus africanus</i> Root Extract. *African Journal of Traditional Complementary and Alternative Medicines*, *5*(1).

Herrera-Calderon, O., Chacaltana-Ramos, L. J., Huayanca-Gutiérrez, I. C., Algarni, M. A., Alqarni, M., & Batiha, G. E. (2021). Chemical Constituents, In Vitro Antioxidant Activity and In Silico Study on NADPH Oxidase of Allium sativum L. (Garlic) Essential Oil. *Antioxidants*, *10*(11), 1844.

Hossain, M. A., Al-Toubi, W. A., Weli, A. M., Al-Riyami, Q. A., & Al-Sabahi, J. N. (2013). Identification and characterization of chemical compounds in different crude extracts from leaves of Omani neem. *Journal of Taibah University for Science*, *7*(4), 181–188.

Ibraheem, O., Oyewole, T. A., Adedara, A., Abolaji, A. O., Ogundipe, O. M., Akinyelu, J., Eze, C. T., Albogami, S., Alotaibi, S. S., Adeyemi, O. S., Batiha, G. E., Alorabi, M., & De Waard, M. (2022). Ackee (Blighia sapida K.D. Koenig) Leaves and Arils Methanolic Extracts Ameliorate CdCl2-Induced Oxidative Stress Biomarkers in Drosophila melanogaster. *Oxidative Medicine and Cellular Longevity*, *2022*, 1–20.

Igbinidu, G., & Nimenibo Uadia, R. (2019). GC-MS analysis, phytochemical screening and in vitro alpha-amylase and alpha-glucosidase inhibitory activities of Vernonia amygdalina root extract and fractions. *J Pharmacogn Phytochem*, *8*(4), 2125-2131.

Igwe, K. K., Madubuike, A. J., Akomas, S. C., Otuokere, I. E., & Ukwueze, C. S. (2016). Studies of the medicinal plant Euphorbia hirta methanol leaf extract phytocomponents by GCMS Analysis. *International Journal of Scientific and Technical Research in Engineering (IJSTRE)*, *1*(4), 9–16.

Ikewuchi, C. C., Ikewuchi, J. C., & Ifeanacho, M. O. (2016). Bioactive phytochemicals in an aqueous extract of the leaves of Talinum triangulare. Food Science & Nutrition, 5(3), 696–701

Ingkaninan, K., Temkitthawon, P., Chuenchom, K., Yuyaem, T., & Thongnoi, W. (2003). Screening for acetylcholinesterase inhibitory activity in plants used in Thai traditional rejuvenating and neurotonic remedies. *Journal of Ethnopharmacology*, *89*(2–3), 261–264

Jain, N. K., Tailang, M., Kumar, S., Chandrasekaran, B., Alghazwani, Y., Chandramoorthy, H. C., Kumar, A., Deshpande, H., Wal, P., Balamurugan, M., & Chidambaram, K. (2022). Appraising the therapeutical potentials of Alchornea laxiflora (Benth.) Pax & K. Hoffm., an underexplored medicinal herb: A systematic review. *Frontiers in Pharmacology*, *13*.

Jana, D., Guchhait, K. C., Ballav, S., Panda, A. K., & Ghosh, C. (2021). Traditional herbal medicine practiced in Plateau-Fringe and Rarh districts of West Bengal, India. In *Springer eBooks* (pp. 1019–1089).

Jash, S. K., Gangopadhyay, A., Sarkar, A., & Gorai, D. (2013). Phytochemical investigation of the hexane extract of stem bark of Peltophorum pterocarpum (DC.). *Der Pharma Chemica*, *5*(5), 49-53.

Jash, S., Singh, R., Majhi, S., Sarkar, A., & Gorai, D. (2014). peltophorum pterocarpum: chemical and pharmacological aspects. International Journal of Pharmaceutical Sciences and Research, 5(1).

Joshi, G., Rawat, Bisht, V., Negi, J., & Singh, P. (2010). Chemical constituents of Asparagus. *Pharmacognosy Reviews/Bioinformatics Trends/Pharmacognosy Review*, *4*(8), 215

Joshua, J. I., Gushit, J. S., Ior, L. D., Ali, A. D., & Dogo, G. A. (2023). Biopestcidal potentials of Erythrophleumafricanum (Benth.) harms stem bark and leaf extracts for the management and control of termites. *Research Square (Research Square)*.

Kain, D., Kumar, S., Vandana, N., Suryavanshi, A., & Arya, A. (2022). FTIR and GCMS analysis of Euphorbia hirtaL. and its In-vitro Antibacterial and Antioxidant Activities. *Indo Global Journal of Pharmaceutical Sciences*, *12*, 104–109.

Karumanchi, N. K., Bhagwan, S. G., Darshana, M., & Bhupendra, K. Mehta. (2016). Phytochemical analysis and antitubercular activity of flower extract of Mangifera indica. International Journal of Pharmaceutical Sciences and Research, 7(8).

Koorbanally, N., Mulholland, D. A., & Crouch, N. (2000). Alkaloids and triterpenoids from Ammocharis coranica(Amaryllidaceae). *Phytochemistry*, *54*(1), 93–97.

Macedo, T., Ferreres, F., Pereira, D. M., Oliveira, A. P., Gomes, N. G., Gil-Izquierdo, Á., Valentão, P., Araújo, L., & Andrade, P. B. (2021). Cassia sieberiana DC. leaves modulate LPS-induced inflammatory response in THP-1 cells and inhibit eicosanoid-metabolizing enzymes. *Journal of Ethnopharmacology*, *269*, 113746.

Maroyi, A. (2018). Albizia adianthifolia: botany, medicinal uses, phytochemistry, and pharmacological properties. *The Scientific World JOURNAL*, *2018*, 1–18.

Mary Helen, P. A., Aswathy, M. R., Deepthi, K. G., Rathi Mol, R., Jaison, J., & Jaya-Sree, S. (2013). Phytochemical analysis and anticancer activity of leaf extract of mangifera indica (kottukonam varika). International Journal of Pharmaceutical Sciences and Research, 43, 823–828.

Masondo, N., Stafford, G., Aremu, A., & Makunga, N. (2019). Acetylcholinesterase inhibitors from southern African plants: An overview of ethnobotanical, pharmacological potential and phytochemical research including and beyond Alzheimer’s disease treatment. *South African Journal of Botany*, *120*, 39–64.

Mazumder, M., & Sarkar, A. K. (2023). Mushrooms as a Source of Enzyme Inhibitors. In *Enzyme Inactivation in Food Processing* (pp. 339-360). Apple Academic Press.

Mensah-Agyei, G. O., Ayeni, K. I., & Ezeamagu, C. O. (2020). GC-MS analysis of bioactive compounds and evaluation of antimicrobial activity of the extracts of Daedalea elegans: A Nigerian mushroom. *African Journal of Microbiology Research*, *14*(6), 204-210.

Mercija, J., & Nevaditha, N. T. (2022). GC-MS analysis of bioactive phytochemicals of gum exudates of Mangifera indica. linn. International Journal of Health Sciences, 1873–1891.

Mfengwana, P. H., & Mashele, S. S. (2020). Medicinal properties of selected asparagus species: a review. *IntechOpen eBooks*.

Mohammed, M., Musa, M. M., Garba, A. M., Adeiza, A. A., & Hanwa, A. (2014). Phytochemical and antimicrobial study on the leaf extracts of Erythrophleum africanum(Caesalpiniaceae). *African journal of biotechnology*, *13*(4), 598–603

Momoh, J. O., & Olaleye, O. N. (2022). Evaluation of Secondary Metabolites Profiling of Ginger (Zingiber officinale Roscoe) Rhizome using GC-MS and Its Antibacterial Potential on Staphylococcus aureus and Escherichia coli. Microbiology Research Journal International, 7–31.

Monehin, J. A., Oriola, A. O., Olawuni, I. J., Odediran, S. A., Ige, O. O., Idowu, T. O., & Ogundaini, A. O. (2024). Cholinesterase Inhibitory Compounds from Peltophorum Pterocarpum Flowers. *Chemistry Africa*, *7*(5), 2899-2906.

Monehin, J. A., Oriola, A. O., Olawuni, I. J., Odediran, S. A., Ige, O. O., Idowu, T. O., & Ogundaini, A. O. (2024). Cholinesterase Inhibitory Compounds from Peltophorum Pterocarpum Flowers. Chemistry Africa.

Mubo, A. S., & Ibukun, O. A. (2015). Medicinal plants used in the treatment of neurodegenerative disorders in some parts of Southwest Nigeria. *African Journal of Pharmacy and Pharmacology*, *9*(38), 956–965.

Mubo, A. S., & Ibukun, O. A. (2015). Medicinal plants used in the treatment of neurodegenerative disorders in some parts of Southwest Nigeria. *African Journal of Pharmacy and Pharmacology*, *9*(38), 956–965.

Mubo, A. S., & Ibukun, O. A. (2015). Medicinal plants used in the treatment of neurodegenerative disorders in some parts of Southwest Nigeria. *African Journal of Pharmacy and Pharmacology*, *9*(38), 956–965.

Mubo, A. S., & Ibukun, O. A. (2015). Medicinal plants used in the treatment of neurodegenerative disorders in some parts of Southwest Nigeria. African Journal of Pharmacy and Pharmacology, 9(38), 956–965.

Naeem, Z., Jabeen, K., & Iqbal, S. (2022). MANAGEMENT OF OCHRATOXIGENIC FUNGI BY PHYTOCHEMICALS OF AMARANTHUS VIRIDIS L. *Journal of Animal and Plant Sciences*, *32*(4).

Nayak, P., & Thirunavoukkarasu, M. (2016). A review of the plant Boerhaavia diffusa: its chemistry, pharmacology and therapeutical potential. *The Journal of Phytopharmacology*, *5*(2), 83–92.

Ndhlala, A. R., Aderogba, M. A., Ncube, B., & Van Staden, J. (2013). Anti-oxidative and cholinesterase inhibitory effects of leaf extracts and their isolated compounds from two closely related Croton species. *Molecules*, *18*(2), 1916-1932.

Noundou, X. S., Krause, R., Van Vuuren, S., Ndinteh, D. T., & Olivier, D. (2016). Antibacterial effects of Alchorneacordifolia (Schumach. and Thonn.) Müll. Arg extracts and compounds on gastrointestinal, skin, respiratory and urinary tract pathogens. *Journal of Ethnopharmacology*, *179*, 76–82.

Ogunlesi, M., Okiei, W., Ofor, E., & Eniola, A. (2019). Medicinal and pesticidal potentials of the constituents of the essential oil from Adenia cissampeloides leaves. *Kemija U Industriji*, *68*(1–2), 7–21.

Ojo, O. A., Ajiboye, B. O., Imiere, O. D., Adeyonu, O., Olayide, I., & Fadaka, A. (2018). Antioxidative Properties of Blighia sapida K.D. Koenig Stem Bark Extract and Inhibitory Effects on Carbohydrate Hydrolyzing Enzymes Associated with Non-Insulin Dependent Diabetes Mellitus. *Pharmacognosy Journal*, *10*(2), 376–383.

Ojo, O. A., Ojo, A. B., Ajiboye, B. O., Olaiya, O., Akawa, A., Olaoye, O., Anifowose, O. O., Idowu, O., Olasehinde, O., & Obafemi, T. (2018). Inhibitory effect of Bryophyllum pinnatum (Lam.) Oken leaf extract and their fractions on α-amylase, α-glucosidase and cholinesterase enzyme. *Pharmacognosy journal*, *10*(3).

Okereke, S., Ikpeazu, O., Nwaogwugwu, C., Ezekwe, A., Nosiri, C., Ukogu, P., Osita, D. M., & Emmanuel, A. (2021). GC-MS analysis of Spondias mombin (Linn) methanol leaf extract. *Int J Biochem Res Rev*, *30*, 1-7.

Okoli, C. O., Akah, P. A., Onuoha, N. J., Okoye, T. C., Nwoye, A. C., & Nworu, C. S. (2008). Acanthus montanus: An experimental evaluation of the antimicrobial, anti-inflammatory and immunological properties of a traditional remedy for furuncles. *BMC Complementary and Alternative Medicine*, *8*(1). [h](https://doi.org/10.1186/1472-6882-8-27)

Okoye, N. N., Nwokoye, J. N., & Okoye, C. (2022). Alstoniaboonei de Wild (Apocynaceae) – A review of its ethnomedicinal uses, phytochemistry and pharmacological activities. *Journal of Current Biomedical Research*, *1*(4), 95–116.

Oyebode, O. A., Erukainure, O. L., Ibeji, C. U., Koorbanally, N. A., & Islam, M. S. (2019). Phytochemical constituents, antioxidant and antidiabetic activities of different extracts of the leaves, stem and root barks of Alstonia boonei: an in vitro and in silico study. *Botany Letters*, *166*(4), 444–456.

Poorna, R. R., Anu, J., & Sathiyajith, J. N. (2022). Phytochemical Profiling, In Vitro Antioxidant Activity of Euphorbia hirta Extracts and In Silico Study Targeting Human Peroxiredoxin 5 Receptor. *Current Trends in Biotechnology and Pharmacy*, *16*(3), 344–356.

Raissa, R., Safitri, A., Beltran, M. a. G., & Aulanni’am, A. (2019). Phytoconstituents Investigation on the Ethanolic Extract of Azadirachta indica var. Indonesian and Philippines. *Journal of Physics Conference Series*, *1374*(1), 012018.

Raudone, L., Vilkickyte, G., Marksa, M., & Radusiene, J. (2024). Comparative Phytoprofiling of Achillea millefolium Morphotypes: Assessing Antioxidant Activity, Phenolic and Triterpenic Compounds Variation across Different Plant Parts. *Plants*, *13*(7), 1043.

Rautela, I., Joshi, P., Thapliyal, P., Pant, M., Dheer, P., Bisht, S., Sinha, V. B., Sundriyal, S., & Sharma, M. D. (2020). Comparative GC-MS analysis of euphorbia hirta and euphorbia milli for therapeutic potential utilities. *Plant Archives*, *20*(2), e-ISSN:2581-6063.

Rehman, N. U., Ansari, M. N., Ahmad, W., & Amir, M. (2022). GC–MS Analysis and In Vivo and Ex Vivo Antidiarrheal and Antispasmodic Effects of the Methanolic Extract of Acacia nilotica. *Molecules*, *27*(7), 2107.

Salehi, B., Gültekin-Özgüven, M., Kırkın, C., Özçelik, B., Morais-Braga, M. F. B., Carneiro, J. N. P., Bezerra, C. F., Da Silva, T. G., Coutinho, H. D. M., Amina, B., Armstrong, L., Selamoglu, Z., Sevindik, M., Yousaf, Z., Sharifi-Rad, J., Muddathir, A. M., Devkota, H. P., Martorell, M., Jugran, A. K., . . . Cho, W. C. (2019). Anacardium Plants: Chemical,Nutritional Composition and Biotechnological Applications. *Biomolecules*, *9*(9), 465.

Salvamani, S., Gunasekaran, B., Shukor, M. Y., Bakar, M. Z. A., & Ahmad, S. A. (2016). Phytochemical investigation, hypocholesterolemic and anti-atherosclerotic effects of Amaranthus viridis leaf extract in hypercholesterolemia-induced rabbits. *RSC Advances*, *6*(39), 32685–32696.

Sharma, R., & Veer, I. Singh. (2021). Identification of phytoconstituents using GC-MS and determination of antimicrobial and antimycobacterial activity of Boerhaavia diffusa L. leaves. *Journal of Pharmaceutical Research International*, *33*(36A), 71–86 *Essential Laboratory Techniques*, *11*(1).

Smelcerovic, A., Lamshoeft, M., Radulovic, N., Ilic, D., & Palic, R. (2009). LC–MS Analysis of the Essential Oils of Achillea millefolium and Achillea crithmifolia. *Chromatographia*, *71*(1–2), 113–116

Son, N. (2019). Genus Erythrophleum: Botanical description, traditional use, phytochemistry and pharmacology. *Phytochemistry Reviews*, *18*(3), 571–599.

Sunday, E. A., & Gift, W. P. (2021). Phytochemistry and antioxidant activity of amaranthus viridis L (Green leaf). *World Journal of Advanced Research and Reviews*, *12*(2), 306–314.

Teshika, J. D., Zakariyyah, A. M., Zaynab, T., Zengin, G., Rengasamy, K. R., Pandian, S. K., & Fawzi, M. M. (2018). Traditional and modern uses of onion bulb (Allium cepaL.): a systematic review. *Critical Reviews in Food Science and Nutrition*, *59*(sup1), S39–S70.

Thomas, B., Soladoye, M., Adegboyega, T., Agu, G., & Popoola, O. (2015). Antibacterial and Anti-Inflammatory Activities of <i>Anacardium occidentale</i> Leaves and Bark Extracts. *Nigerian Journal of Basic and Applied Science*, *23*(1), 1.

Vasudevan, V., Karthikeyan, A., Rajamuthu, T. P., Sarkar, A., Paranjothi, G., Krishnan, N., Sakthivelu, M., Velusamy, P., Anbu, P., & Raman, P. (2023, April 25). *Bioactive Metabolites from Germinated Cajanus cajan (L.) Millsp. Seeds after Treating with Different Concentrations of Salicylic Acid*.

Vilela, C., Santos, S. A., Oliveira, L., Camacho, J. F., Cordeiro, N., Freire, C. S., & Silvestre, A. J. (2013). The ripe pulp of Mangifera indica L.: A rich source of phytosterols and other lipophilic phytochemicals. Food Research International, 54(2), 1535–1540.

Wang, J., Du, Y., Jiang, L., Li, J., Yu, B., Ren, C., Yan, T., Jia, Y., & He, B. (2024). LC- MS/MS-based chemical profiling of water extracts of Moringa oleifera leaves and pharmacokinetics of their major constituents in rat plasma. Food Chemistry X, 23, 101585.

Wang, S., Cao, J., Deng, J., Hou, X., Hao, E., Zhang, L., Yu, H., & Li, P. (2021). Chemical characterization of flavonoids and alkaloids in safflower (Carthamus tinctorius L.) by comprehensive two-dimensional hydrophilic interaction chromatography coupled with hybrid linear ion trap Orbitrap mass spectrometry. *Food Chemistry X*, *12*, 100143.

Wesołowska, A., Jadczak, D., & Grzeszczuk, M. (2011). *CHEMICAL COMPOSITION OF THE PEPPER FRUIT EXTRACTS OF HOT CULTIVARS Capsicum annuum*

Yunus, F. T., Suwondo, A., & Martini, N. (2021). Phytochemical Compound of Garlic (Allium sativum) as an Antibacterial to Staphylococcus aureus Growth. *IOP Conference Series Materials Science and Engineering*, *1053*(1), 012041.
